# Doped Highly Crystalline Organic Films: Toward High‐Performance Organic Electronics

**DOI:** 10.1002/advs.202003519

**Published:** 2021-01-27

**Authors:** Michael F. Sawatzki, Hans Kleemann, Bahman K. Boroujeni, Shu‐Jen Wang, Joern Vahland, Frank Ellinger, Karl Leo

**Affiliations:** ^1^ Dresden Integrated Center for Applied Physics and Photonic Materials (IAPP) Technische Universität Dresden Noethnitzer Str. 61 Dresden 01187 Germany; ^2^ Chair of Circuit Design and Network Theory (CCN) Technische Universität Dresden Helmholtz Str. 18 Dresden 01069 Germany; ^3^ Center for Advancing Electronics Dresden (cfaed) Technische Universität Dresden Helmholtz Str. 18 Dresden 01069 Germany

**Keywords:** crystals, diode, high‐frequency, high‐mobility, organic electronics, rubrene

## Abstract

Today's organic electronic devices, such as the highly successful OLED displays, are based on disordered films, with carrier mobilities orders of magnitude below those of inorganic semiconductors like silicon or GaAs. For organic devices such as diodes and transistors, higher charge carrier mobilities are paramount to achieve high performance. Organic single crystals have been shown to offer these required high mobilities. However, manufacturing and processing of these crystals are complex, rendering their use outside of laboratory‐scale applications negligible. Furthermore, doping cannot be easily integrated into these systems, which is particularly problematic for devices mandating high mobility materials. Here, it is demonstrated for the model system rubrene that highly ordered, doped thin films can be prepared, allowing high‐performance organic devices on almost any substrate. Specifically, triclinic rubrene crystals are created by abrupt heating of amorphous layers and can be electrically doped during the epitaxial growth process to achieve hole or electron conduction. Analysis of the space charge limited current in these films reveals record vertical mobilities of 10.3(49) cm^2^ V^−1^ s^−1^. To demonstrate the performance of this materials system, monolithic pin‐diodes aimed for rectification are built. The $f_{3db}$ of these diodes is over 1 GHz and thus higher than any other organic semiconductor‐based device shown so far. It is believed that this work will pave the way for future high‐performance organic devices based on highly crystalline thin films.

## Graphical abstract

Heat‐induced crystalization of rubrene films on substrates is combined with doping via vacuum co‐evaporation to realize thin‐films with exceptional vertical mobility. p‐ and n‐type doping is realized, allowing for free stacking of individual layers and subsequently device structures with great complexity. Exemplary rectifier circuits based on pin‐diodes feature record‐breaking performance, operating above 1 GHz.

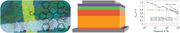

Power‐efficient and high‐performance flexible thin‐film electronic devices would offer a multitude of novel applications. In particular, the maximum switching speed has been identified as the main figure of merit, and operation above the GHz^[^ ^1^ ^]^ threshold is expected to enable new applications, such as analog mixer circuits for wireless communication based on low‐cost flexible electronics. In this regard, much progress has been achieved in the last years in exploring and optimizing organic semiconductors toward the key material performance parameter, that is, charge carrier mobility. Record values for field‐effect mobility of 40 cm^2^ V^−1^ s^−1^ have been shown by Takeya et al.^[^ ^2^ ^]^ based upon single‐crystal rubrene and 10 cm^2^ V^−1^ s^−1^ for thin‐film C_8_‐BTBT by Haase et al.^[^ ^3^ ^]^ An alternative strategy to increase the switching frequency of electronic devices, aside from an increase in charge carrier mobilities, is the reduction of the defining device dimension (e.g., channel length in transistors). While in lateral devices, the reduction of channel length can only be done using costly structuring methods, vertical device designs offer the possibility to easily realize ultra‐short devices via the control of layer thickness. This idea has been successfully proven in devices like vertical diodes and transistors.^[^ ^4^, ^5^, ^6^, ^7^ ^]^ Most concepts used to realize high‐mobility organic material systems feature a significant increase in complexity, incompatible with standard procedures. Thus, most advanced device designs are based on comparably simple and low‐performance material systems, while most devices made with high‐mobility materials are based on simple device geometries and designs. Hence, combining the concept of vertical device geometries with high mobility materials is the logical next step to reach ultra high frequency devices.

The highest charge carrier mobilities in organic semiconductors have been measured in furnace grown single‐crystals.^[^ ^2^ ^]^ However, it is practically impossible to use these crystals for thin‐film electronics. Not only because the growth method and fragility of these crystals require manual handling, but because thin‐film processing is not viable for loosely adhering crystals. Thus, one of the cost‐saving advantages of organic electronics would be nullified. Alternatively, crystalline thin‐films with high charge carrier mobility can be grown by methods like shear‐coating from solution^[^ ^3^ ^]^ or heat‐induced crystallization.^[^ ^8^ ^]^ Unfortunately, the high mobility in such thin‐films does not necessarily translate to equivalent improvement in device performance (e.g., switching frequency) which is mainly due to the influence of contact resistance.^[^ ^9^ ^]^ The most effective way for a reduction of contact resistance is doping.^[^ ^10^ ^]^ However, bulk doping^[^ ^11^ ^]^ is traditionally difficult in highly crystalline (^[^ ^12^, ^13^ ^]^) or solution‐processed systems, due to the disturbance caused by the dopant during growth and the reduced order. Thus, finding procedures for effective doping of crystalline films is essential to enable fast devices based on high mobility materials and optimized geometries.

The single‐crystal form of rubrene is difficult to utilize in thin‐film electronics, thus, other means to produce crystals—preferably as layers—have been developed. An early approach by Park et al.^[^ ^14^ ^]^ and later Lee et al.^[^ ^15^ ^]^ is based upon the recrystallization of previously amorphous films of rubrene by rapid heating. The general process is depicted at the top of **Figure** **1**b. Different crystal polymorphs are formed depending on the heating temperature, time, and properties of the surface of the substrate. The focus in most publications is to achieve the orthorhombic polymorph in the form of flat extended platelets (see Figure S10, Supporting Information). These layers feature lateral field‐effect mobilities of up to 4 cm^2^ V^−1^ s^−1^.^[^ ^8^ ^]^ They are thus well suited for lateral charge transport. However, since most organic semiconductor crystals feature a strong anisotropy, high mobility in lateral direction does not necessarily translate into high vertical mobility.^[^ ^16^ ^]^ According to data from Lee et al.,^[^ ^17^ ^]^ the *c*‐axis of crystalline rubrene is oriented perpendicular to the substrate in all known thin‐film polymorphs. A decrease in molecular distance of rubrene has been shown to increase the mobility,^[^ ^18^ ^]^ due to a more favorable molecular orientation and thus overlap of molecular orbitals. Although we expect significantly lower lateral mobilities, due to its dendritic growth mode (see Figure 1d), the triclinic crystal phase (*c* = 11.87 Å^[^ ^19^ ^]^)—formed at lower heating temperatures—should be more suitable for vertical transport than the orthorhombic phase (*c* = 25.91 Å) due to the denser packing along the c‐axis. The molecular packing of this triclinic phase is depicted in Figure 1a.

**Figure 1:**
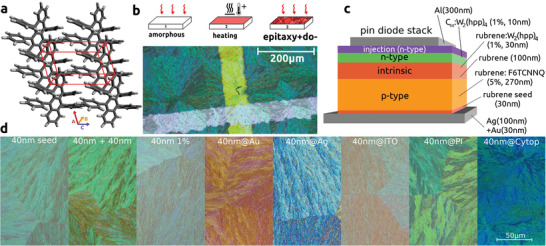
a) unit cell of triclinic thin‐film rubrene, b) depiction of growth process via heating of amorphous thin‐film (resulting in seed layer) and consecutive epitaxy (top), active device area of rubrene‐based diode, c) material stack of rectifier diode (doping in weight percent), d) polarization micrograph of triclinic rubrene crystals on various surfaces.

Here, we study the triclinic crystal phase of surface crystallized rubrene as thin layers grown by heat treatment and consecutive epitaxial growth. One advantage of this crystal phase is its simpler and more robust growth process, compared to the other polymorphs. Precise control of temperature^[^ ^17^ ^]^ or a complicated surface treatment^[^ ^8^ ^]^ are not necessary. Fabrication of devices is possible on a wide variety of substrates and surfaces, ranging from SiO_2_‐coated Si‐wafers and glass, over electrode materials like Au, Ag, and ITO to polymers such as polyimide for use as a flexible substrate or even Cytop. We study the effect of p‐ and n‐doping on the conductivity of these layers and measure the vertical mobility. As an application utilizing the very high vertical mobilities offered by this material system, we present ultra high frequency organic rectifier pin‐diodes which enable efficient rectification well above the GHz threshold and hence could be used in novel applications such as energy harvesting in radio‐frequency identification (RFID) tags or analog mixer circuits for wireless communication based on low‐cost flexible electronics.

Surface doping (p‐ and n‐type) is commonly used for organic thin‐films to reduce injection barriers in field‐effect transistors^[^ ^20^ ^]^ and OLEDs.^[^ ^21^ ^]^ In contrast, bulk‐doping of organic semiconductor crystals is more difficult to realize but would enable efficient charge transport over longer distances, ambipolar devices, and complementary circuits. Beyond the improvement of charge carrier transport and injection, bulk doping is a valuable tool to influence important device parameters like capacitances, leakage currents, charge carrier balance in OLEDs, or onset voltages.

Epitaxy onto crystalline seed layers has been presented by Verret et al.^[^ ^22^ ^]^ This process of seed layer formation and consecutive epitaxy is schematically shown in Figure 1b. We integrate co‐evaporation of molecular dopants into this epitaxy step. A more detailed description of the procedure is given in the Supporting Information. Based on polarized microscopy and atomic force microscopy measurements, we conclude that the structure of the triclinic films does not change when dopants are integrated into the bulk. In contrast to the orthorhombic crystal phase that crystallizes in large flat platelets, the triclinic polymorph grows in spherulitic dendrites. The triclinic phases are significantly more robust against variation of growth parameters like temperature, seed thickness, and surface properties than the platelet films. They can be grown on standard substrates like Si‐wafers and glass as well as on polymers like polyimide and Cytop and are thus better suited for future flexible electronics than rubrene platelet films or even single‐crystals.

Weak doping (<2 wt.%) is possible even for the seed layer itself, without any visible change to the crystals. Higher doping concentrations in the seed film reduce the yield of crystallization and decrease the average grain diameter. However, since the impact of seed‐doping on the *I–V* characteristics is small (see Figure S2, Supporting Information), we used undoped seeds for all further experiments. We suggest diffusion of free majority charge carrier into the undoped seed as a possible mechanism. The diffusion length for excitons in rubrene has been found to be longer than 200 nm in orthorhombic crystals.^[^ ^22^ ^]^ Considering the large carrier mobility in the triclinic layers, a (majority) hole diffusion length of 40 nm seems possible. One distinction to other organic crystalline systems^[^ ^23^, ^24^ ^]^ is the high doping ratios that can be achieved. While the seed can be doped safely to at most 2 wt.% F6‐TCNNQ, epitaxially grown layers did not show any change in polarization microscopy until at least 10 wt.%. Structural measurements did not show any change in crystallinity at 2 wt.% doping (see Figure S11).

**Figure** **2**a shows the *I–V* characteristics of 400 nm metal‐semiconductor‐metal devices in the triclinic crystal phase doped with the p‐type dopant F6‐TCNNQ in the epitaxial part of the stack (see Figure S1, Supporting Information). Layers below 250 nm show a strong tendency for short‐circuits. We attribute this to the rough surface of these films. The initial roughness created via the seed crystallization remains during the consecutive epitaxy (see Figure S14, Supporting Information).

**Figure 2:**
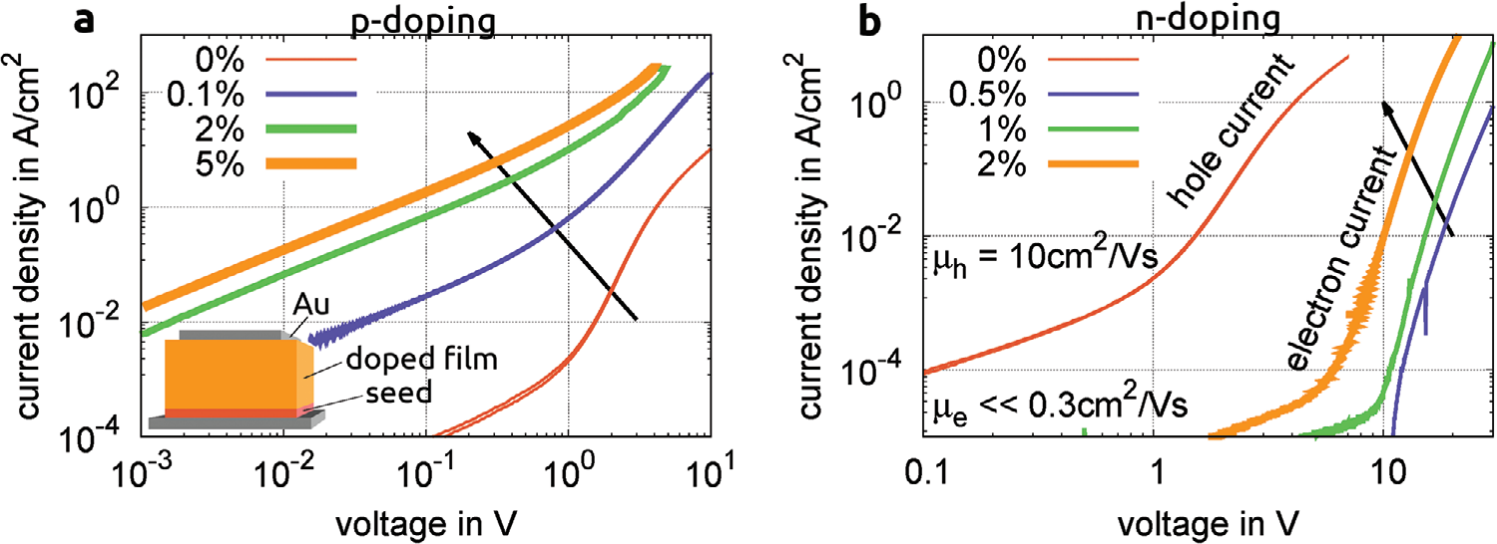
*I*–*V* characteristics of metal‐semiconductor‐metal devices of triclinic rubrene thin‐films with various doping concentrations (in weight percent). a) p‐doping with F6‐TCNNQ, b) n‐doping with $W_{2}{\left(\mathrm{hpp}\right)}_{4}$, active area: 100μm×100μm, Au electrodes, total thickness: 400 nm, no additional injection doping (stack as inset, for details see Figure S1, Supporting Information).

Free carriers are present even in the undoped films due to unavoidable impurities introduced during the manufacturing (see Part D, Supporting Information). The resulting current is carried by higher‐mobility holes.^[^ ^25^ ^]^ The current density increases strongly once doping is introduced, rising by up to two orders of magnitude for 0.1 wt.% F6‐TCNNQ. This is partly due to increased conductivity and partly due to improved injection. A higher doping concentration increases the conductance further and results in a more linear behavior of the *I–V* characteristic. The increase in free charge carrier density is further backed by a Mott–Schottky analysis of differently doped Schottky diodes (see Part D, Supporting Information). Due to the resulting very high conductance, current densities of several hundreds of A cm^−2^ are reached without any damage to the layers.

For n‐doping, $W_{2}{\left(\mathrm{hpp}\right)}_{4}$ is used (Figure 2b), with the same undoped seed and co‐evaporation epitaxy that is used for the p‐doped films. Polarization microscopy and AFM‐images (see Figure S13, Supporting Information) do not indicate any change in crystal structure. The *I–V* curves of these layers feature a sudden shift to higher voltages and consecutively lower conductance than even the undoped layers. Higher doping concentrations show a partial recovery in conductance. We assume that the initial (weak) doping depletes the film of free holes which are caused by impurity doping. The increase in density of free electrons can not compensate for the loss of holes since the resulting electron‐only conduction is less efficient due to lower electron mobility in rubrene. Even transport in single‐crystals along the most favorable axis shows electron mobility two orders of magnitude lower than corresponding hole mobility ($\mu_{e}=$0.3 cm^2^ V^−1^ s^−1^).^[^ ^25^ ^]^ The partial recovery of conductance can be explained by generation of additional free electrons. Additionally, injection barriers might play a role for the lower doped devices, as well as a lower doping efficiency (ionisation potential $E_{I}$($W_{2}{\left(\mathrm{hpp}\right)}_{4}$) = 3.5 eV,^[^ ^26^ ^]^ LUMO(rubrene) = 3.0 eV^[^ ^27^ ^]^). Overall, effective doping of p‐ and n‐type can be achieved, although p‐doped layers have a significantly higher resulting conductance.

To quantify the possible performance for vertical devices, a hole conduction‐based space charge limited current (SCLC) analysis is performed. 600 nm, 800 nm, and 1000 nm of intrinsic rubrene is sandwiched between Au electrodes of varying active area and 40 nm of injection layer on both sides doped with 5 wt.% F6‐TCNNQ (see Figure S1, Supporting Information). The *I–V* curves are symmetrical for positive and negative bias indicating effective injection. At high currents, a distinct $I\propto V^{2}$ dependence emerges (see inset **Figure** **3**). Assuming an SCLC and using the Mott–Gurney relation

(1) $$j=\frac{9}{8}\varepsilon_{r}\varepsilon_{0}\mu \frac{V^{2}}{L^{3}},$$

the vertical mobility of holes in these layers is deduced to $\mu_{h}=$10.3(49) cm^2^ V^−1^ s^−1^, using an $\varepsilon_{r}=2.6$.^[^ ^28^ ^]^ A detailed explanation of the extraction method is given in the Part C, Supporting Information. The variation between individual samples is larger for the thinner layers, presumably due to a larger variation in effective thickness caused by the larger roughness of these films. The measured mobility is significantly higher than the current record for vertical mobility in organic materials found in vertically aligned P3HT.^[^ ^29^ ^]^ Additionally, the vertical mobility in these triclinic layers is larger than the lateral mobility measured in orthorhombic films (4 cm^2^ V^−1^ s^−1^^[^ ^8^ ^]^) and the vertical (*c*‐axis) mobility measured in furnace‐grown single‐crystals (3.5 cm^2^ V^−1^ s^−1^^[^ ^28^ ^]^). In contrast, the lateral mobility in these triclinic films is lower than for their orthorhombic counterparts, although still comparable to other standard transistor materials. We found hole mobilities in the range of $1\times 10^{-2}\;{\mathrm{cm}}^{2}\;V^{-1}\;s$ in field‐effect measurements (see Part E, Supporting Information). The strong anisotropy in charge carrier mobility cannot be explained by the difference in electronic transport along different molecular axis alone. It is a result of the dendritic structure of these crystals. While in the vertical direction (*c*‐axis), the films show high order and crystallinity, transport parallel to the substrate is characterized by constant trapping at grain boundaries between adjacent dendrites, resulting in a severely decreased effective lateral charge carrier mobility.

**Figure 3:**
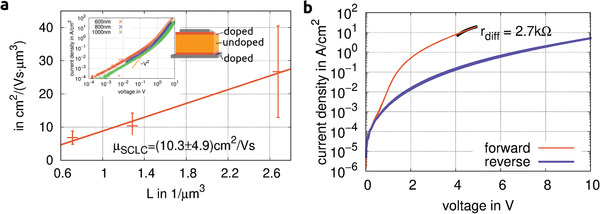
SCLC measurement: a) Fits of mobility according to the Mott–Gurney law over a set of ten different active areas per intrinsic layer thickness based on *I*–*V* characteristics (inset) of intrinsic triclinic rubrene thin‐films of various thickness sandwiched between doped injection layers and Au electrodes (stack as inset, for details see Figure S1, Supporting Information), b) *I–V*‐characteristic of rectifier diode based on triclinic rubrene thin‐films. Device stack according to Figure 1c (The measurement is performed on a device outside of the rectifier circuit).

Nevertheless, organic semiconductor devices that feature current transport in vertical or mixed direction can greatly benefit from these high vertical mobilities. Several transistor designs utilize vertical transport to generate short channels,^[^ ^30^ ^]^ reaching record switching frequencies^[^ ^7^ ^]^ and current densities^[^ ^31^ ^]^ with comparably low mobility materials. We present pin‐diodes oriented toward rectification of ultra high frequency signals as a possible application. Ultra high frequency diodes are a key component for flexible, active organic RFID technology operating in the GHz‐regime.^[^ ^32^ ^]^ They are essential for power harvesting, RF switching, and signal demodulation. Since diodes can operate at significantly higher frequencies than even the fastest organic transistor,^[^ ^7^ ^]^ they are ideal to pre‐process ultra high frequency signals for a slower transistor‐based circuit.

The maximum frequency $f_{\max}$ at which an incoming signal with the voltage ΔV can still be processed by a film of thickness L can be estimated via the inverse transit time^[^ ^33^ ^]^:

(2) $$f_{\max}=\frac{\mu \Delta V}{2\pi L^{2}}.$$

Thus, high average mobility across a signal diode is essential for fast operation.

The stack of the rectifier diodes is shown in Figure 1c, and the corresponding active area in Figure 1b. The devices we present here are pin‐diodes, in contrast to Schottky‐diodes which were tested too. All tested pin diodes could operate at higher frequencies than the Schottky diodes based on the same material system (see Figure S7, Supporting Information). This result is the opposite of what is typical for inorganic diodes. We attribute this property to a longer reverse recovery time and consecutive increased reverse leakage current of the Schottky‐diodes. It is caused by a lack of recombination within the intrinsic layers, which can be counteracted with the addition of n‐doped films. Since the conductivity of the p‐doped side is significantly larger than the n‐doped layers, the majority of the stack is made up of p‐doped material. Properties of the diode can be tuned by varying the thickness of the intrinsic and n‐doped layers. The current is mainly limited by the intrinsic film, the corresponding depletion layers, and—to a lesser extent—by the n‐doped film. We can achieve on/off ratios of up to $10^{5}$ (see Figure S6, Supporting Information) and current densities in the 100 A cm^−2^‐regime in constant power mode and kA cm^−2^ pulsed (see Figure S4, Supporting Information). We add a thin layer of n‐doped $C_{60}$ on the n‐doped side to improve electron injection.

Figure 3b shows the *I–V* characteristics of a diode optimized for maximum frequency rectification. A corresponding capacitance measurement is shown in Figure S16, Supporting Information, showing the formation of the charge depletion zone and its voltage dependence. Since there is no indication of strong dispersion, we assume the measurement of the capacitance at 1 MHz is comparable to the high‐frequency regime. The maximum switching frequency can be estimated from the capacitance and differential resistance at the operation point:

(3) $$f_{\text{RC}}=\frac{2\pi }{r_{\text{diff}}C}=3.7\;\mathrm{GHz},$$

using the DC properties of the device. This quantity describes the limit for a particular device in a specific configuration, in contrast to Equation (2) that relates to the capabilities of the material itself. Thus, Equation (2) always gives an estimate for an upper limit for the highest technically achievable switching frequency, neglecting, for example, series resistance and effects related to stray capacitance.

A half‐wave rectifier circuit is a simple way to assess the dynamic capabilities of a diode. The circuit we used is shown in **Figure** **4**a. Parasitic R‐L‐C effects are mostly neglected in other publications^[^ ^4^ ^]^ but can affect the circuit in the GHz regime. We minimized parasitic influences by miniaturization of the circuit and integration of compensation capacitors $C_{m1}$. Figure 4c shows the waveform of the input and output signal at 1 MHz. The output voltage of the rectifier decreases with frequency as shown in Figure 4d due to the finite switching speed of the diode. Further details regarding the measurement setup are given in the Supporting Information.

**Figure 4:**
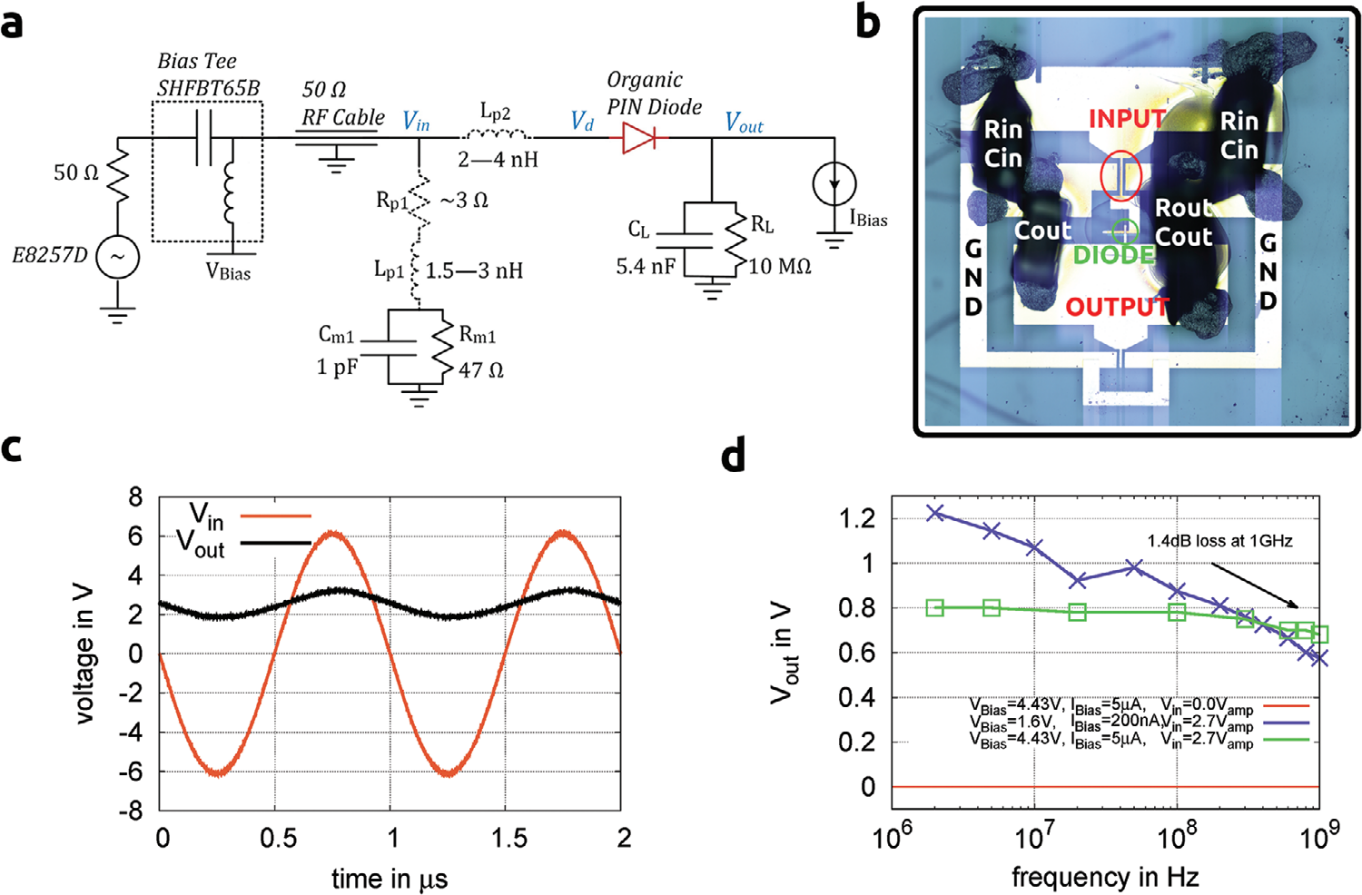
a) Circuit diagram of the measurement setup. Parasitic resistance and inductance are shown in dashed line. b) rectifier circuit including organic diode and setup components. c) Input and output waveforms at 1 MHz and 6 V amplitude, $C_{L}$=10 pF, $I_{\text{Bias}}$=0 μA, $V_{\text{Bias}}$=0 V. d) Rectified DC output voltage as a function of frequency at 2.7 V amplitude and $C_{L}$=5.4 nF. Active area: 50μm×50μm $V_{\text{Bias}}=$DC‐voltage of the diode bias, $I_{\text{Bias}}=$DC‐current of the diode bias, $V_{\text{in}}$=amplitude of the sinusoidal signal, according to circuit diagram (a).

The device has a loss of only 1.4 dB at 1 GHz with 5 μA DC current and approximately 10 m AC current passing through it. This is 3.5 dB less loss compared to the previously fastest organic diode reported by Kang et al.^[^ ^34^ ^]^ (additional values for comparison are given in the Table S1, Supporting Information). This can be ascribed to the significantly higher vertical charge carrier mobility of the rubrene films used here compared to pentacene.

Thermal effects are commonly neglected in literature. However, the AC Joule‐heating of the diode is significant and can be described as $P=2\pi^{2}R_{d}{\left(C_{d}V_{d}f\right)}^{2}$, where $R_{d}$ is the series resistivity of the bulk of n‐doped and p‐doped layers. To measure reproducibly at high frequencies, we introduced a DC‐bias such that the diode can be driven at a lower AC amplitude and power. However, $V_{\text{Bias}}$ is tuned to have zero $V_{\text{out}}$ at zero AC input, therefore the DC‐biasing is not influencing the rectification directly. The input signal $V_{d}$ at the diode is stable across the entire frequency range (see Figure S8, Supporting Information).

With an $f_{3dB}$ over 1 GHz, our devices are truly the first published fully organic rectifier diodes operating in the GHz‐regime. Utilizing equation 2, with *L* = 140 nm as the relevant thickness (intrinsic and n‐doped layers are limiting due to the weak doping) and the vertical mobility measured previously, the transition frequency of this type of diode could become as large as 37 GHz with ΔV=4.5 V. There is still room for improvement since optimization of injection and doping is possible.

It is especially worth noting that these frequencies are reached despite the absence of proper encapsulation of the diode (Cytop is used to protect against water) and the tendency of rubrene to degrade in the presence of oxygen.^[^ ^35^ ^]^ Devices show degradation during the process of measurement and the destruction of the device at 1 GHz can be partly attributed to accelerated heat‐induced oxidization. Although challenging due to the miniaturization necessary for high‐frequency measurements, it can be assumed that even higher frequencies might be reached once effective encapsulation against oxygen is implemented.

We present a method for the growth of highly crystalline layers of rubrene utilizing a rapid heating method to produce seed crystals and consecutive epitaxial growth by thermal evaporation. We use the lesser‐known triclinic phase due to its superior vertical mobility compared to orthorhombic polymorphs of rubrene. Vertical hole mobilities of 10.3(49) cm^2^ V^−1^ s^−1^ set a new record for organic materials. The growth process for the triclinic polymorph is robust and can be performed on a variety of substrates and surfaces. Processing on polyimide is possible and allows for future flexible applications. Additionally, p‐doping and n‐doping can be integrated into the crystals, increasing conductivity and enabling complex device stacks. As an example, we present monolithic pin‐diodes optimized for ultra high frequency rectification. The test circuit reaches a milestone for organic electronics with operation frequencies above 1 GHz.

The flexibility in processing and doping allows expanding the use of these layers to other applications that can benefit from the large vertical mobilities, especially diode‐based circuits and vertical transistors. However, due to the comparably low charge carrier mobility of the electrons in rubrene, the limiting factor for the diode devices is electron transport. This can be partly counteracted via the design of the devices and used circuits. A particular interest can be seen in literature in regards to native n‐type organic single crystals.^[^ ^36^, ^37^ ^]^. Nevertheless, a material system that offers comparably high performance while offering similar ease of manufacturing and handling is yet to be found. This is important not only for ambipolar devices—like pin diodes—where both carrier types are present and the slowest process usually dominates but also for applications where transistors are focused since many circuits require complementary transistor technology for optimum performance.

## Experimental Section

##### Sample Preparation

Devices are either built on glass or Si wafers with a size of 25 mm × 25 mm. Substrates are cleaned in acetone, ethanol, isopropanol, and de‐ionized water. Each of the substrates is treated in piranha solution for 15 min to generate a clean and hydrophilic surface. They are rinsed in de‐ionized water and dried with nitrogen.

Layers are deposited via thermal evaporation in vacuum under a base pressure of $1\times 10^{-8}$ mbar. The evaporation rate of the seed has no influence on the further process. After deposition of the bottom metal electrode (30–40 nm) and the first amorphous layer of rubrene (30–40 nm), samples are transferred to a nitrogen glovebox, without exposure to air. Heat treatment takes place on a pre‐heated hotplate at 130${}^{\circ }$C, for 15 min. If needed, additional layers are added using co‐evaporation of rubrene and dopant with the same vacuum deposition at rates between range 0.5 and 3 Å s^−1^, depending on the doping concentration. Electrodes and semiconductor are structured using shadow masks. Active areas for conductivity and SCLC‐measurements range from 50μm×50μmto150μm×150μm. Diodes for high‐frequency rectifiers have an active area of 50μm×50μm. Devices used for conductivity measurements have a total thickness of 400 nm. The initial seed is undoped. No further doping aside the given bulk doping is introduced at the electrodes.

Electrical DC‐measurements are performed using a Keithley 2600 SMU and capacitance measurements with an HP 4284A in a nitrogen atmosphere. Micrographs were taken with a Nikon Eclipse LC100 PL/DS polarization microscope. The AFM measurements are performed with an AIST‐NT Combiscope1000. Semiconductor material is provided by TCI.

Performance and properties of stack‐wise identical devices vary significantly. This is to a large extent the result of the randomness intrinsic to the crystallization method resulting in different absolute thicknesses, different grain orientations, and different grain sizes for each of the devices. It is worth noting, however, that the process is not yet optimized for highest reproducibility. Many process steps influence the procedure, such as the comparably small substrate size, non‐uniform heat distribution on the hotplate, manual timing of the heating step, or convection currents and gas flow within the glovebox during crystallization.

##### Measurements and Circuit Simulations

To minimize parasitic resistance, inductance, and capacitance (*R*–*L*–*C*) in the signal path, and to push the *L*–*C* self‐resonance frequencies away from the measurement bandwidth, that is, to keep the signal amplitude $V_{d}$ as constant as possible up to GHz range, we build the rectifier circuit directly on the substrate of the diode using 1.6 mm long SMD components. The parasitic $L_{p1}$ increases the voltage at $V_{\text{in}}$ at high frequencies, therefore $C_{m1}$ is added to compensate for this effect. Parasitic interconnect resistance $R_{p1}$ is measured to be around 3 Ω. For this reason, $R_{m1}$=47 Ω is used to have a total matching of 50 Ω. Since components are mounted manually, the exact value of $L_{p1,2}$ is not known, and therefore in the simulation shown in Figure S8, Supporting Information they are swept over the expected range estimated using extraction tools. The dielectric constant of polymers usually decreases with frequency, therefore the diode capacitance is also swept from 0.4 to 0.8 pF. Diode's series resistance should be above 10 Ω, but has a small impact on $V_{d}$. The deviation of $V_{d}$ is less than 9% at 1 GHz when all parameters are swept. This deviation would be as high as 20% without $C_{m1}$. In order to further decrease the $L_{p1,2}$, two parallel sets of *R*–*C* are placed at the input and output, that is, in Figure 5 we have $R_{\text{in}}$=94 Ω, $C_{\text{in}}=0.5$ pF, and $C_{\text{out}}$=2.7 nF. The diode is first biased at a fixed current of 200 nA or 5 μA, then $V_{\text{Bias}}$ is tuned to have $V_{\text{out}}$=0 V at AC input $V_{\text{in}}$=0 V. In this way, we also have $V_{\text{out}}$=0 V at the frequency of infinity for non‐zero AC $V_{\text{in}}$. By adding DC biasing, a lower $V_{d}$ amplitude is needed for measuring the rectifier performance, resulting in lower Joule heating at high frequencies. The losses in the RF cable and bias tee are measured and compensated by first connecting the cable to the Rohde and Schwarz FSV 7 GHz signal analyzer, then tuning the signal generator Keysight E8257D to deliver exactly 18.7 dBm power on each measurement point. Total harmonic distortion at $V_{\text{in}}$ is measured to be less than 1 %.

## Authors Contribution

Experiments regarding the material system, growth of crystals on various substrates, doping experiments, SCLC analysis and the fabrication of the diodes have been performed by M.S. S.J.W. and J.V. contributed to measurements shown in the Supporting Information. These works were supervised by H.K. and K.L. The HF measurement setup and its analysis were performed by B.K.B. and supervised by F.E. All authors reviewed the manuscript.

## Conflict of Interest

The authors declare no conflict of interest.

## Supporting information

Supporting Information
