# Supplementary material for: Doped Highly Crystalline Organic Films: Toward High‐Performance Organic Electronics
Source: Adv Sci (Weinh). 2021 Jan 27;8(6):2003519. doi: 10.1002/advs.202003519 (PMC7967074; doi:10.1002/advs.202003519)
Supplement: Supplementary file 1 — Supporting Information [file ADVS-8-2003519-s001.pdf]

© 2021 Wiley-VCH GmbH

ADVANCED SCIENCE Open Access

Supporting Information

for *Adv. Sci.,* DOI: 10.1002/advs.202003519

Doped Highly Crystalline Organic Films: Toward High-Performance Organic Electronics#

Michael F. Sawatzki, Hans Kleemann, Bahman K. Boroujeni, Frank Ellinger, and Karl Leo*

Doped Highly Crystalline Organic Films: Towards High-performance Organic Electronics

Michael F. Sawatzki
Hans Kleemann
Bahman K. Boroujeni
Frank Ellinger
Karl Leo

Dr. Michael Franz Sawatzki
Dresden Integrated Center for Applied Physics and Photonic Materials (IAPP), Technische Universität Dresden, Noethnitzer Str. 61, 01187 Dresden, Germany Email Address: michael.sawatzki@tu-dresden.de
Dr. Hans Kleemann
Dresden Integrated Center for Applied Physics and Photonic Materials (IAPP), Technische Universität Dresden, Noethnitzer Str. 61, 01187 Dresden, Germany Email Address: hans.kleemann1@tu-dresden.de
Dr. Ing. Bahman Kheradmand Boroujeni
Chair of Circuit Design and Network Theory (CCN), Technische Universität Dresden, Helmholtz Str. 18, 01069 Dresden, Germany Center for Advancing Electronics Dresden (cfaed), Technische Universität Dresden, Helmholtz Str. 18, 01069 Dresden, Germany Email Address: bahman.kheradmand_boroujeni@tu-dresden.de
Prof. Dr. sc. techn. habil. Frank Ellinger
Chair of Circuit Design and Network Theory (CCN), Technische Universität Dresden, Helmholtz Str. 18, 01069 Dresden, Germany Center for Advancing Electronics Dresden (cfaed), Technische Universität Dresden, Helmholtz Str. 18, 01069 Dresden, Germany Email Address: frank.ellinger@tu-dresden.de
Prof. Dr. Karl Leo
Dresden Integrated Center for Applied Physics and Photonic Materials (IAPP), Technische Universität Dresden, Noethnitzer Str. 61, 01187 Dresden, Germany Center for Advancing Electronics Dresden (cfaed), Technische Universität Dresden, Helmholtz Str. 18, 01069 Dresden, Germany Email Address: karl.leo@tu-dresden.de

Keywords: *organic electronics, diode, rubrene, crystals, high-frequency, high-mobility*

Today's organic electronic devices, such as the highly successful OLED displays, are based on disordered films, with carrier mobilities orders of magnitude below those of inorganic semiconductors like silicon or GaAs. For organic devices such as diodes and transistors, higher charge carrier mobilities are paramount to achieve high performance. Organic single crystals have been shown to offer these required high mobilities. However, manufacturing and processing of these crystals are complex, rendering their use outside of laboratory-scale applications negligible. Furthermore, doping cannot be easily integrated into these systems, which is particularly problematic for devices mandating high mobility materials.
Here, we demonstrate for the model system rubrene that highly ordered, doped thin films can be prepared, allowing high-performance organic devices on almost any substrate. Specifically, triclinic rubrene crystals are created by abrupt heating of amorphous layers and can be electrically doped during the epitaxial growth process to achieve hole or electron conduction. Analysis of the Space Charge Limited Current in these films reveals record vertical mobilities of $(10.3 \pm 4.9)\,\mathrm{cm^2\,V^{-1}\,s^{-1}}$. To demonstrate the performance of this materials system, we build monolithic pin-diodes aimed for rectification. The f_{3dB} of these diodes is over 1 GHz and thus higher than any other organic semiconductor-based device shown so far. We believe that our work will pave the way for future high-performance organic devices based on highly crystalline thin films.

1 Sample Preparation

Devices are either built on glass or Si wafers with a size of 25 mm × 25 mm. Substrates are cleaned in acetone, ethanol, isopropanol, and de-ionized water. Each of the substrates is treated in piranha solution for 15 min to generate a clean and hydrophilic surface. They are rinsed in de-ionized water and dried with nitrogen.
Layers are deposited via thermal evaporation in vacuum under a base pressure of $1 \cdot 10^{-8}$ mbar. The evaporation rate of the seed has no influence on the further process. After deposition of the bottom metal

electrode (30 nm to 40 nm) and the first amorphous layer of rubrene (30 nm to 40 nm), samples are transferred to a nitrogen glovebox, without exposure to air. Heat treatment takes place on a pre-heated hotplate at 130 °C, for 15 min. If needed, additional layers are added using co-evaporation of rubrene and dopant with the same vacuum deposition at rates between $0.5\,\text{Å}\,\text{s}^{-1}$ to $3\,\text{Å}\,\text{s}^{-1}$, depending on the doping concentration. Electrodes and semiconductor are structured using shadow masks. Active areas for conductivity and SCLC-measurements range from 50 µm × 50 µm to 150 µm × 150 µm. Diodes for high-frequency rectifiers have an active area of 50 µm × 50 µm. Devices used for conductivity measurements have a total thickness of 400 nm. The initial seed is undoped. No further doping aside the given bulk doping is introduced at the electrodes.
Electrical DC-measurements are performed using a Keithley 2600 SMU and capacitance measurements with an HP 4284A in a nitrogen atmosphere. Micrographs were taken with a Nikon Eclipse LC100 PL/DS polarization microscope. The AFM measurements are performed with an AIST-NT Combiscope1000. Semiconductor material is provided by TCI.
Performance and properties of stack-wise identical devices vary significantly. This is to a large extent the result of the randomness intrinsic to the crystallization method resulting in different absolute thicknesses, different grain orientations, and different grain sizes for each of the devices. It is worth noting, however, that the process is not yet optimized for highest reproducibility. Many process steps influence the procedure, such as the comparably small substrate size, non-uniform heat distribution on the hotplate, manual timing of the heating step, or convection currents and gas flow within the glovebox during crystallization.

2 Statistical Analysis

The data shown in Figure 2 corresponds to the raw-data measured in an IV-measurement. Only the active area was taken into account to transform the current into a current density. In this graph, the curves from devices with an active area of 100 µm × 100 µm are shown as an example from sets of devices ranging from 50 µm × 50 µm to 150 µm × 150 µm. Although the absolute conductivity in these measurements varies significantly (we assume this to be caused by the randomness of the crystallization process), the general shape of the IV-curves is the same for devices featuring the same doping concentration. The trend between different doping concentrations is the same for all active areas.
The data collected from graph 3a stems from sets of at least ten devices (varying active area) per device thickness. In particular, devices featuring only a thin intrinsic film showed several devices with short circuits. These were subsequently discarded and not used in the fitting. The values gained are thus averages from ten devices per film thickness, while the whiskers correspond to 1σ around the value. The errors of the individual measurements are used in the fitting. The resulting $r^2 = 0.98$. The error of the final value for the mobility is derived from the maximum and minimum slope that is compatible with the error bars given by the data points at each thickness.
Figure 3b represents one of the diodes that is integrated into the rectifier circuit. Except for the normalization to the active area, the data is unchanged.
Figure 4 shows the data of one rectifier diode circuit. The frequency behavior can be replicated with different devices. However, since fixing the smd components for the surface to the glass substrate is difficult, only a few circuits are made in total.
Data shown in supplementary information are unfiltered and unchanged, aside from a normalization to the area or thickness. Explanations for box-plots are given in the figure captions. Whiskers of all measurements correspond to a 1σ interval.

3 Measurements and Circuit Simulations

To minimize parasitic resistance, inductance, and capacitance (R-L-C) in the signal path, and to push the L-C self-resonance frequencies away from the measurement bandwidth, i.e. to keep the signal amplitude V_d as constant as possible up to GHz range, we build the rectifier circuit directly on the substrate

of the diode using 1.6 mm long SMD components. The parasitic L_{p1} increases the voltage at V_{in} at high frequencies, therefore C_{m1} is added to compensate for this effect. Parasitic interconnect resistance R_{p1} is measured to be around 3 Ω. For this reason, R_{m1}=47 Ω is used to have a total matching of 50 Ω. Since components are mounted manually, the exact value of $L_{p1,2}$ is not known, and therefore in the simulation shown in Figure S8 they are swept over the expected range estimated using extraction tools. The dielectric constant of polymers usually decreases with frequency, therefore the diode capacitance is also swept from 0.4 pF to 0.8 pF. Diode's series resistance should be above 10 Ω, but has a small impact on V_d. The deviation of V_d is less than 9 % at 1 GHz when all parameters are swept. This deviation would be as high as 20 % without C_{m1}. In order to further decrease the $L_{p1,2}$, two parallel sets of R-C are placed at the input and output, i.e. in Figure 5 we have R_{in}=94 Ω, C_{in}=0.5 pF, and C_{out}=2.7 nF. The diode is first biased at a fixed current of 200 nA or 5 µA, then V_{Bias} is tuned to have V_{out}=0 V at AC input V_{in}=0 V. In this way, we also have V_{out}=0 V at the frequency of infinity for non-zero AC V_{in}. By adding DC biasing, a lower V_d amplitude is needed for measuring the rectifier performance, resulting in lower Joule heating at high frequencies. The losses in the RF cable and bias tee are measured and compensated by first connecting the cable to the Rohde&Schwarz FSV 7 GHz signal analyzer, then tuning the signal generator Keysight E8257D to deliver exactly 18.7 dBm power on each measurement point. Total harmonic distortion at V_{in} is measured to be less than 1 %.

4 Justification for the Use of Mott-Gurney Law

Several criteria have to be fulfilled in our devices to justify the SCLC analysis used to calculate vertical charge carrier mobilities from the Mott-Gurney law:
1. A purely hole-based transport is well justified, since the p-type injection doping is enhancing hole injection and impeding electron injection [1]. Furthermore, the charge carrier mobility of holes in rubrene is significantly larger than for electrons. Thus, even for unselective electrodes, the dominant transport type is almost entirely hole-based.
2. The clear kink in the IV curve (see Figure 3) highlighting the transition from linear to V^2-behavior, indicates that as soon as the device enters the SCLC regime, the injected charge carrier density outweighs the intrinsic density significantly. Thus, charge carriers are entering via one electrode and exciting via the others, while effectively no intrinsic conductivity is present. Due to the selective electrodes and the high HOMO-LUMO gap of rubrene, virtually no electrons are present and hence holes move through the device without recombination! Ohmic injection is guaranteed by the doped layers at the contacts showing almost perfectly Ohmic behavior up to 100 A cm^{-2} (in the IV measurements) which is above the highest current density we reach in the SCLC analysis (cf. Fig 2 and Fig 3)!
3. The material composition and structure of the rubrene is – aside from the doped injections layer – identical throughout the film, which justifies the assumption of constant dielectric constant.
Due to the extended density of states in most organic semiconductors, density and field-dependence of the charge carrier mobility are commonly observed in organic semiconductors. However, rubrene is an exception in this regard since its crystalline nature leads to a narrow DOS. Thus, charge carrier mobility does typically not dependent much on the charge carrier density or strength of electric fields [2].
4. (compare to comment 3) Rubrene shows very little disorder and hence posses a narrow density of states. Also trap states are not common to rubrene since band-like transport has been observed even at comparably low charge carrier densities [3]. Regardless of these aspects, our SCLC analysis also provides clear evidence for trap-free transport since we found a V^2-dependence and not a more general power law such as $V^{2+\kappa}$. Furthermore, our SCLC analysis is rigorous since three different layer thicknesses are measured. Instead of calculating the resulting charge carrier mobility from the slope of the Mott-Gurney law of each of those devices individually, the thickness dependence is used to ensure SCLC-behavior. Thus, Figure 3b shows the thickness-weighted mobility $(\frac{\mu}{L^3})$ over the inverse thickness $\frac{1}{L^3}$. The linearity of this graph proves the appropriate $\frac{1}{L^3}$-dependence, while the slope of the linear fit gives in the corresponding charge carrier mobility.
5. The conductivity of the rubrene films increases in the low voltage regime by more than five orders of

magnitude once doping is introduced (see Figure 2). This suggests that the density of free charge carriers in the doped films is approximately five orders of magnitude higher than in the undoped films (depending on the behavior of the mobility)! Consequently, in a pip device, the vast majority of charge carriers in the intrinsic layer is injected from the doped layers and carried through the intrinsic region only by the electric field. The increase in hole density is additionally supported by the Mott-Schottky analysis we added to the SI (from $3 \cdot 10^{14}\,\mathrm{cm}^{-3}$ to $>1 \cdot 10^{17}\,\mathrm{cm}^{-3}$).
6. The added injection layers created via bulk doping of both sides of the undoped films realize ohmic contacts. The effectiveness of the doping itself is evident from the doping experiments shown in Figure 2a. For the high doping concentration of 5 wt.% used for the injection, even bulk films show almost completely ohmic behavior over several orders of magnitude. Ohmic injection is further supported by the symmetry of the IV curve shown by the SCLC devices, even at high bias voltages. This translates into no voltage drop over the electrodes and thus no field.
We thus conclude that all conditions with regards to SCLC behavior and extraction of charge carrier mobilities via the Mott-Gurney law are met.

5 Charge Carrier Density

We performed a Mott Schottky analysis on Schottky diodes based on doped triclinic rubrene crystals (see Figure S1). Seeds are undoped for all devices. The charge carrier density is extracted from a fit of the inverse squared capacitance over the bias voltage, measured at 100 kHz. Here, values for 0.1 wt.%, 0.5 wt.%, and 5 wt.% are shown. Error bars originate from devices with different active areas and different thicknesses of the corresponding triclinic part. Thus, the charge carrier density steadily increases with additional doping, explaining the further increase in conductivity with doping.
We can estimate the density of charge carriers (likely holes) in the intrinsic film from the crossing point of the linear and squared regime in the SCLC measurement shown in Figure 3 to $3 \cdot 10^{14}\,\mathrm{cm}^{-3}$ according to [4]. It is thus several orders of magnitude lower than the lowest doping concentration measured here, explaining the strong increase in conductivity.

6 Lateral Charge Carrier Mobility

The main focus here is on the vertical transport on doped crystalline rubrene films and corresponding devices. However, it can be insightful to study the lateral transport additionally to assess the viability of the system for devices with a predominantly lateral (OFETs) or mixed (advanced transistor designs) mode of carrier transport. Here, a simple bottom-gate, top-contact configuration OFET is chosen as a vehicle. Devices are built in a global-gate configuration on a p-doped Si-wafer, semiconductor is added over the entire substrate area, and Au-electrodes (40 nm) are structured via shadow-masking. The gate insulator is a combination of 100 nm SiO_2 and 50 nm of Cytop. We found that adding a layer of 30 nm of intrinsic rubrene on top of the seed (40 nm) prior to electrode deposition increases the performance. Based on the AFM images (see Figure S12), we assume the individual strands of the dendrites are poorly connected in lateral direction. Adding material fills these gaps to a certain degree.
Channel width is 1000 µm and channel length varies between 50 µm to 200 µm. No strong change in the normalized performance can be seen from devices with different channel lengths. Injection resistance is hence not a limiting factor in these measurements and can be neglected compared to the channel resistance. We found an average charge carrier mobility of $(0.012 \pm 0.007)\,\mathrm{cm}^2\,\mathrm{V}^{-1}\,\mathrm{s}^{-1}$. The high performance shown by these films in vertical direction is thus not present in the lateral direction to the same degree. This is a result of the strongly dendritic growth of these triclinic crystals. While the films are highly crystalline along the vertical direction (c-axis), the same high degree of crystallinity is only present within a single dendrite in lateral direction. Charge carriers can only travel freely until they reach the next dendritic arm, where scattering or trapping occurs. As can be seen from the AFM images of Figure S12, this results in a free path of around 50 nm to 200 nm. As a consequence, the average (effective)

charge carrier mobility in lateral direction is significantly smaller than the molecular charge carrier mobility. Nevertheless, the performance shown is still high in the scope of organic semiconductors in general. A use in devices with transport in multiple directions is still promising.

7 Comments to Equation 2:

The equation is based on the vertical transit time t of charge carriers with the average speed v through a film of organic material with the thickness d:

$$t = \frac{d}{\overline{v}}. \tag{1}$$

Assuming a constant mobility and a homogeneous field through the film:

$$\overline{v} = \overline{\mu E} = \frac{\mu_f E}{2} + \frac{\mu_r E}{2} = \frac{\mu_f E}{2} = \frac{\mu_f V}{2d}, \tag{2}$$

with μ_f as the forwards hole mobility and $\mu_r = 0$ as the effective reverse mobility. Translating the transit time into a frequency, gives equation 2

$$f_{max} = \frac{\mu \Delta V}{2\pi d^2}. \tag{3}$$

We chose, $\Delta V = V_{Bias} - V_{out} + \frac{V_d}{\pi}$ =4.5 V as the corresponding voltage across the diode to show the potential of these films compared to the already high frequencies for the devices measured.

8 Speed of Signal Rectification of Organic Diodes

The frequency response of the rectifier circuit is limited by the slowest component. By design, this is commonly the diode. At low frequencies, the output voltage of the rectifier remains constant with frequency, given the smoothing capacitor is large enough. However, once a certain device-given frequency is reached several possible processes might limit the speed of operation. A finite turn-on time results in the forward regime not being reached within one cycle. The capacitor is thus not charged. Furthermore, a finite reverse recovery time (the time needed to switch the diode from forward to reverse operation) can lead to a significant drain of the accumulated charges during the reverse cycle. Regardless of the precise mechanism, the rectified output voltage decreases at sufficiently high frequencies. Publications featuring organic electronics use different metrics to define the point of this transition and thus the frequency performance of devices. A direct comparison of values is hence difficult.
Many publications show values for the cut-off frequency – f_0 – which is defined as the maximum frequency for which any signal can still be rectified. Although valid for comparison of devices in itself, this value is of little use in real applications. The common standard in electrical engineering[5] is the cut-off frequency f_{3dB}, defined by the frequency at which the output **power** is halved compared to the low-frequency regime, which corresponds to the output power being reduced to $\frac{1}{\sqrt{2}}$.
However, even when values for f_{3dB} are given, proper extraction is often not clear. Wrong readings are presented and resonances at the input side of the circuit are rarely accounted for, leading to an over-estimation of the real performance. Additionally, the AC power introduced into the system grows with frequency. Early onset in the reduction of the device performance can thus be masked via the increase of conductivity from heating. A property that is sometimes given is the "f_{3dB} in terms of voltage", defined as the frequency at which the output **voltage** is halved. However, since the unit decibel is defined in terms of power quantities, these supposedly "f_{3dB} frequencies are actually values for "f_{6dB}.

References

**parameter**	definition	impact and use
f_0	$f\,(V_{out}=0)$	The maximum frequency of rectification activity.
$f_{\frac{V}{2}}$ sometimes "f_{3dB} in terms of voltage"	$f\left(V_{out}=\frac{V_{dc}}{2}\right)$	The frequency at which output voltage drops by a factor of $\frac{1}{2}$ compared to low frequencies, i.e. output power drops by a factor of 4.
f_C	$f_C=\frac{\mu\Delta V}{2\pi L^2}$	Frequency derived from the transit time, describing the speed at which a charge carrier can pass a layer of thickness L of a material with the charge carrier mobility of μ.
f_{3dB} in terms of power	$f\left(P_{out}=\frac{P_{out}}{2}\right)$ $f\left(V_{dc}=\frac{V_{out}}{\sqrt{2}}\right)$	The frequency at which output voltage drops by a factor of $\frac{1}{\sqrt{2}}$ compared to low frequencies, i.e. output power drops by a factor of 2. This is the most common figure of merit for the bandwidth in electrical engineering.

Table 1: Performance parameters for rectifier circuits.

**year**	material system	claimed benchmark frequency	reference
2016	small molecule (pentacene)	1.24 GHz (f_{3dB})*	[6]
2012	small molecule (C_{60}, pentacene)	1 GHz (f_0)	[7]
2015	printed polymer (PTAA)	>10 MHz	[8]
2012	small molecule (pentacene)	>5 MHz	[9]
2017	solution processed (IDT-BT)	68 MHz to 125 MHz (f_C)	[10]
2005	small molecule (pentacene)	50 MHz	[11]

Table 2: Organic rectifiers.

References

[1] N. B. Kotadiya, H. Lu, A. Mondal, Y. Ie, D. Andrienko, P. W. M. Blom, G.-J. A. H. Wetzelaer, *Nature Materials* **2018**, *17*, 329.

[2] F. Bussolotti, J. Yang, T. Yamaguchi, K. Yonezawa, K. AU Sato, M. Matsunami, K. Tanaka, Y. Nakayama, H. Ishii, N. Ueno, S. Kera, *Nature Communications* **2017**, *8*, 173.

[3] Y. Mei, P. J. Diemer, M. R. Niazi, R. K. Hallani, K. Jarolimek, C. S. Day, C. Risko, J. E. Anthony, A. Amassian, O. D. Jurchescu, *Proceedings of the National Academy of Sciences* **2017**, *114*, 33 E6739.

[4] S. C. Jain, W. Geens, A. Mehra, V. Kumar, T. Aernouts, J. Poortmans, R. Mertens, M. Willander, *Journal of Applied Physics* **2001**, *89*, 7 3804.

[5] B. Razavi, *Design of Analog CMOS Integrated Circuits*, McGraw-Hill Education Ltd, **2000**.

[6] C.-m. Kang, J. Wade, S. Yun, J. Lim, H. Cho, J. Roh, H. Lee, S. Nam, D. D. C. Bradley, J.-S. Kim, C. Lee, *Advanced Electronic Materials* **2016**, *2*, 2 1500282.

[7] H. Kleemann, S. Schumann, U. Jörges, F. Ellinger, K. Leo, B. Lüssem, *Organic Electronics* **2012**, *13*, 6 1114 .

[8] P. Heljo, C. Schmidt, R. Klengel, H. Majumdar, D. Lupo, *Organic Electronics* **2015**, *20* 69 .

[9] G. Gutierrez-Heredia, V. H. Martinez-Landeros, F. S. Aguirre-Tostado, P. Shah, B. E. Gnade, M. Sotelo-Lerma, M. A. Quevedo-Lopez, *Semiconductor Science and Technology* **2012**, *27*, 8 085013.

[10] S. G. Higgins, T. Agostinelli, S. Markham, R. Whiteman, H. Sirringhaus, *Advanced Materials* **2017**, *29*, 46 1703782.

[11] S. Steudel, K. Myny, V. Arkhipov, C. Deibel, S. De Vusser, J. Genoe, P. Heremans, *Nature Materials* **2005**, *4*, 8 9.

[12] P. K. Koech, A. B. Padmaperuma, L. Wang, J. S. Swensen, E. Polikarpov, J. T. Darsell, J. E. Rainbolt, D. J. Gaspar, *Chemistry of Materials* **2010**, *22*, 13 3926.

[13] F. Albert Cotton, P. Huang, C. A. Murillo, X. Wang, *Inorganic Chemistry Communications* **2003**, *6*, 2 121 .

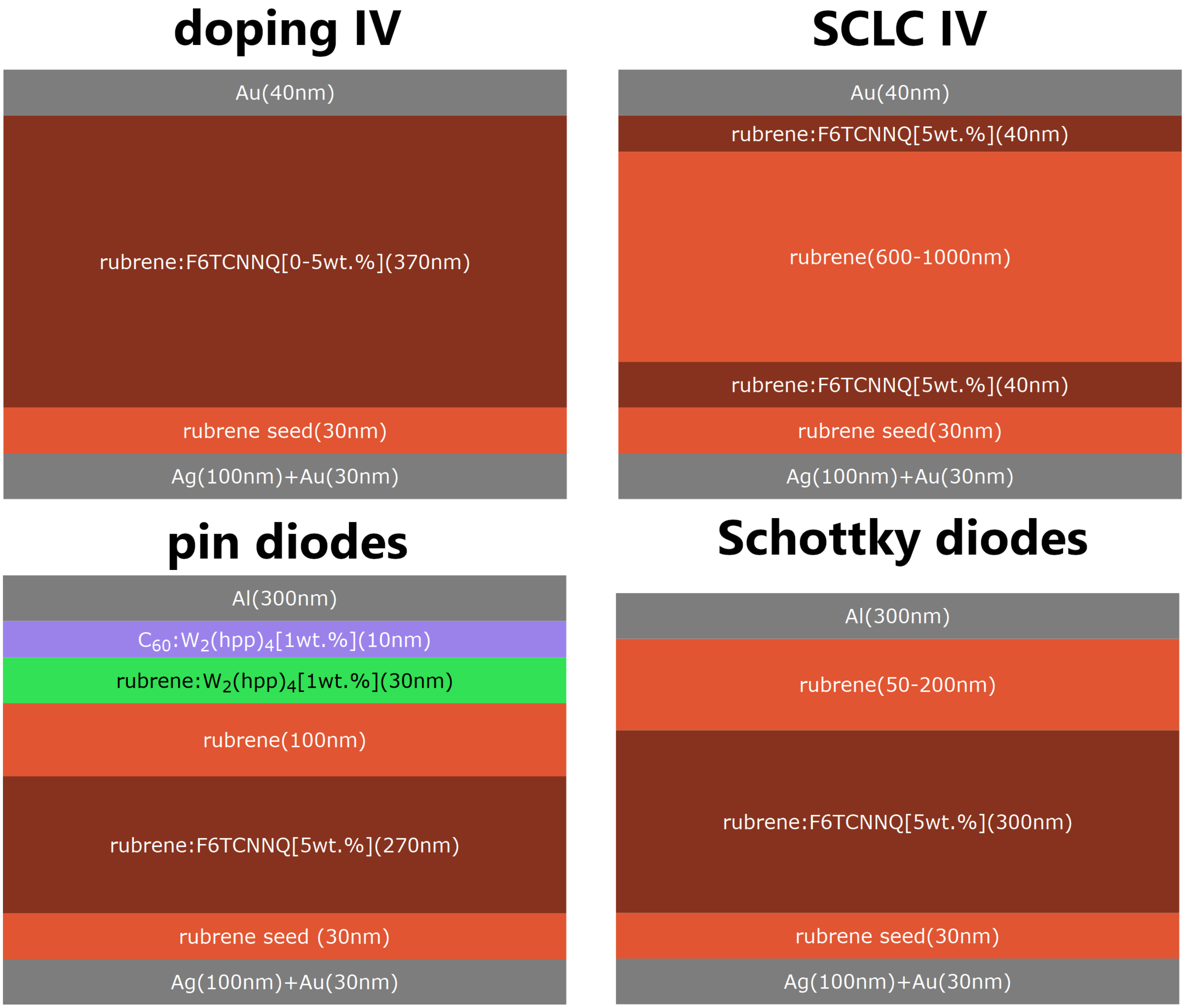

Figure 1: Stack configuration if all three types of devices built and analyzed.

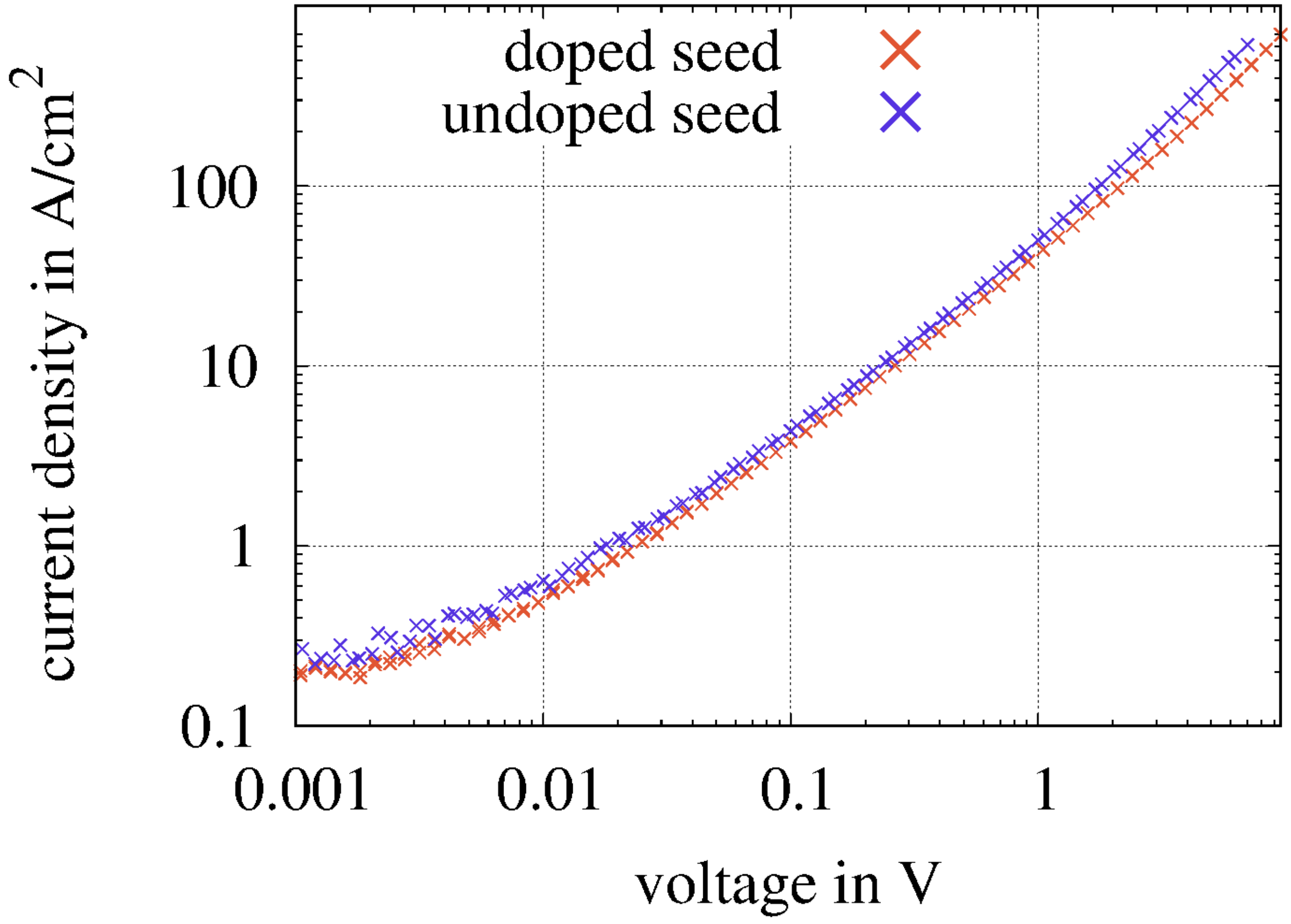

Figure 2: MSM(Metal-Semiconductor-Metal)-sandwich structure of in total 400 nm rubrene: The bulk of the films is doped with 0.5 wt.% $C_{60}F_{36}$. One samples has a doped seed with 0.5 wt.% $C_{60}F_{36}$, the other seed is undoped.

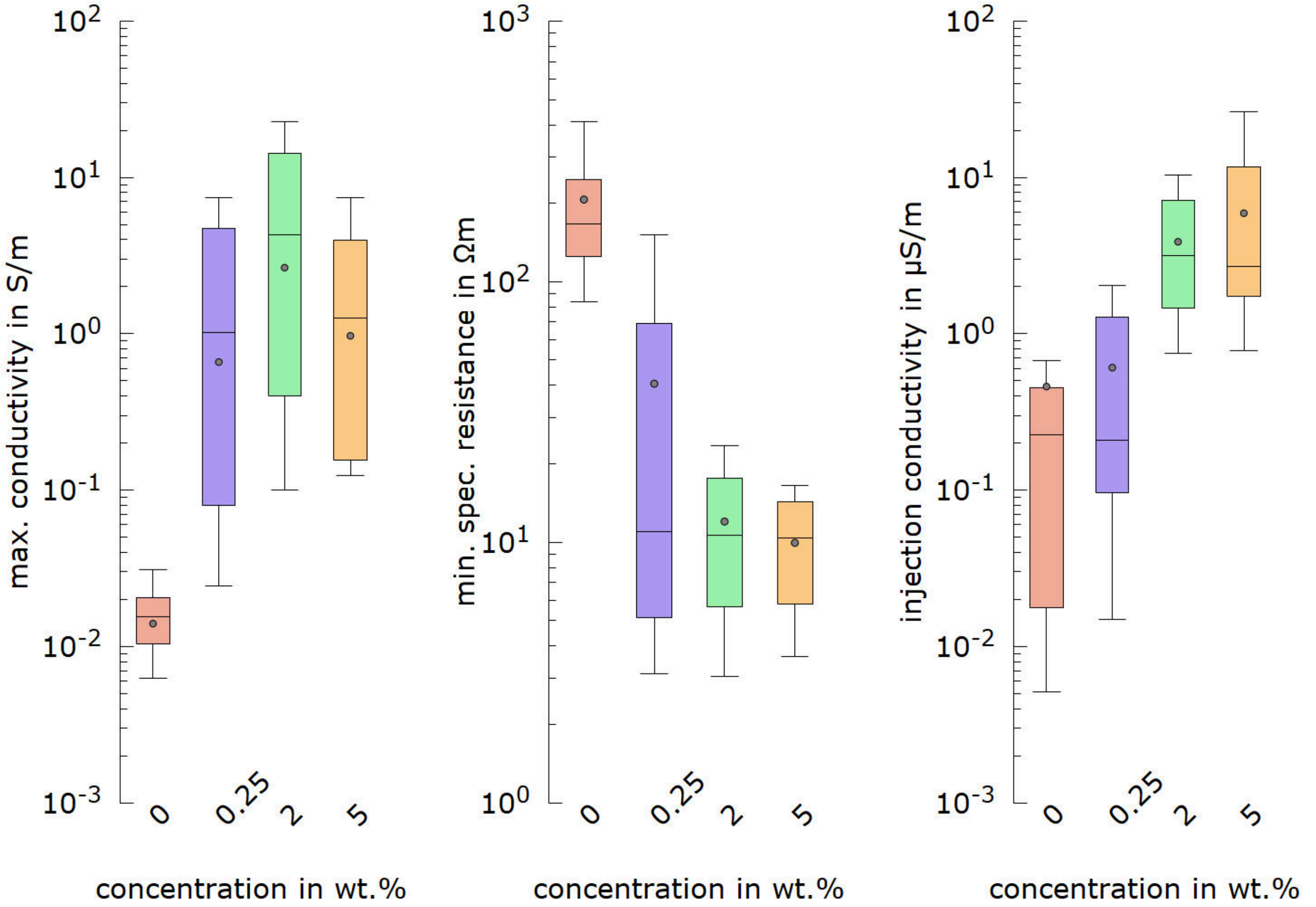

Figure 3: Statistics of different devices of the set shown in Figure 2a: Variation in maximum conductivity in the measured voltage range ($\sigma^{\max} = \max\left(\frac{\partial j}{\partial V}\right)$), lowest specific resistance from $R^{\min}$ $\min\left(\frac{U}{I}\right)$, and conductivity in the low voltage regime indicative of injection (defined as $\left(\frac{\partial j}{\partial V}\right)$ at low voltages). The data is generated from 20 to 25 devices per doping concentration. Line in the middle of the boxplot indicates median value while the boxes represent the adjacent quartiles. Circles indicate the average value. Figure 2 shows a representative device for each doping concentration that reflects the general behavior of the corresponding set the best.

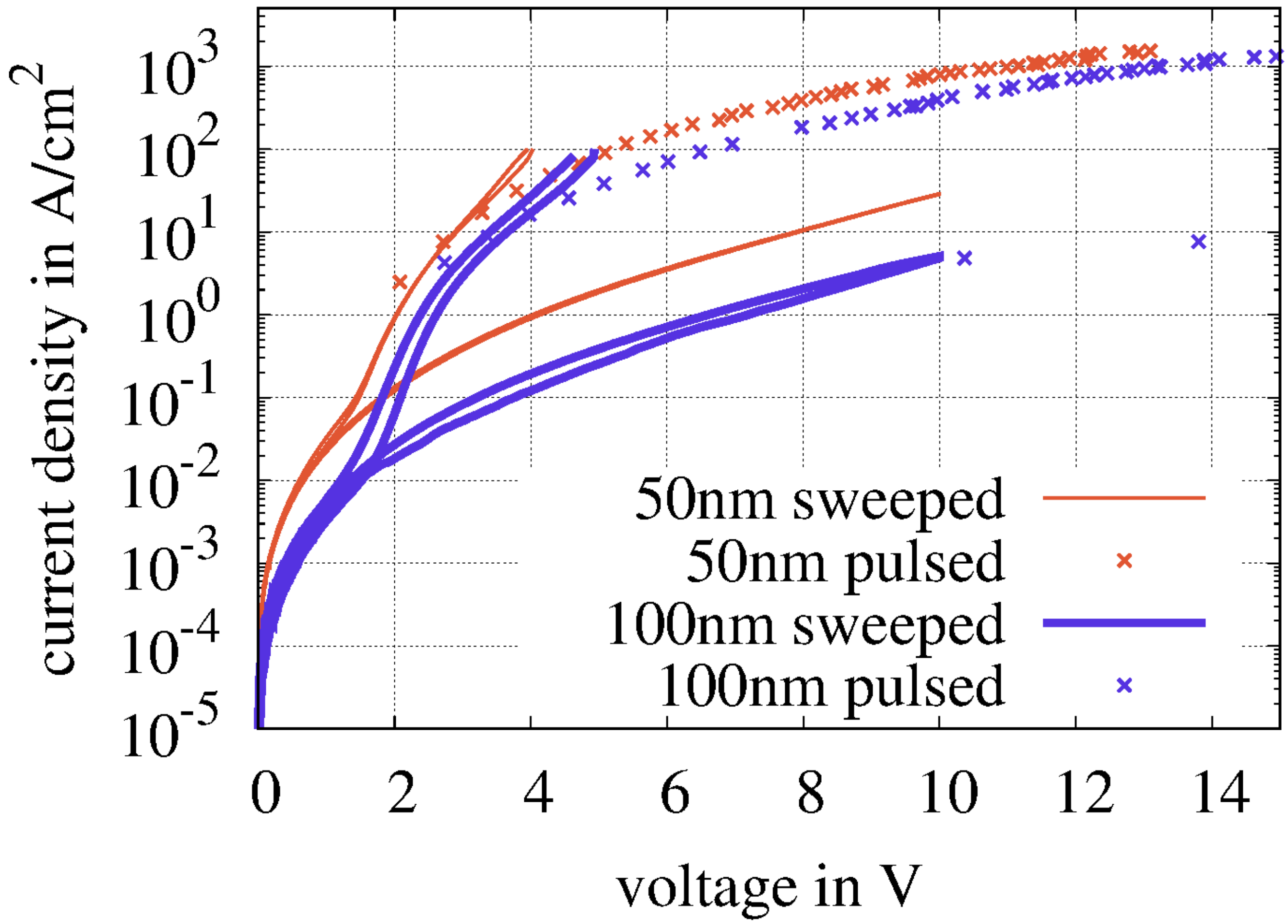

Figure 4: Schottky-diodes aimed at maximum current density: Au-bottom electrode, 30 nm undoped seed, 300 nm rubrene doped with 5 wt.% F6-TCNNQ, 50 nm/100 nm of intrinsic rubrene, Al top-electrode. Pulsed measurements are performed with a hp 8110a pulse generator with 100 µs pulses at 111 Hz repetition. Current is measured through a 47 Ω series resistor.

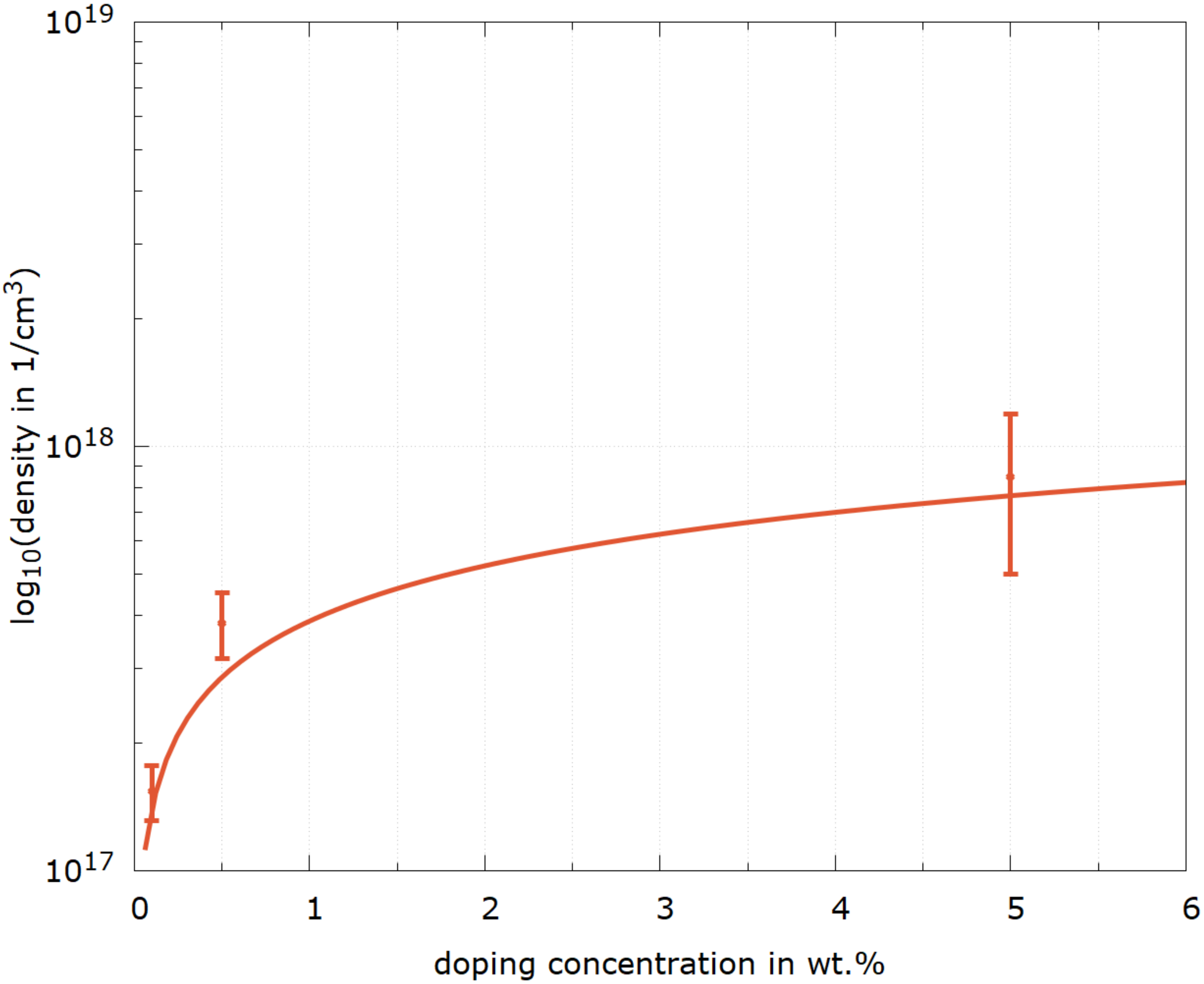

Figure 5: Free hole densities extracted from Mott-Schottky analysis of Schottky diodes based on triclinic rubrene films. An ohmic Au electrode is connected to a layer of p-doped rubrene of 300 nm thickness doped at different doping concentrations. Different thickness of intrinsic films are added, followed by an Al electrode for the top contact. Capacitances for the fit are measured at 100 kHz using an Agilent 4284A LCR meter. The error bars are the standard distribution of devices with different active areas and different thickness of the intrinsic film. The added line does not correspond to any physical relationship but is merely a guide to the eye for intermediate values.

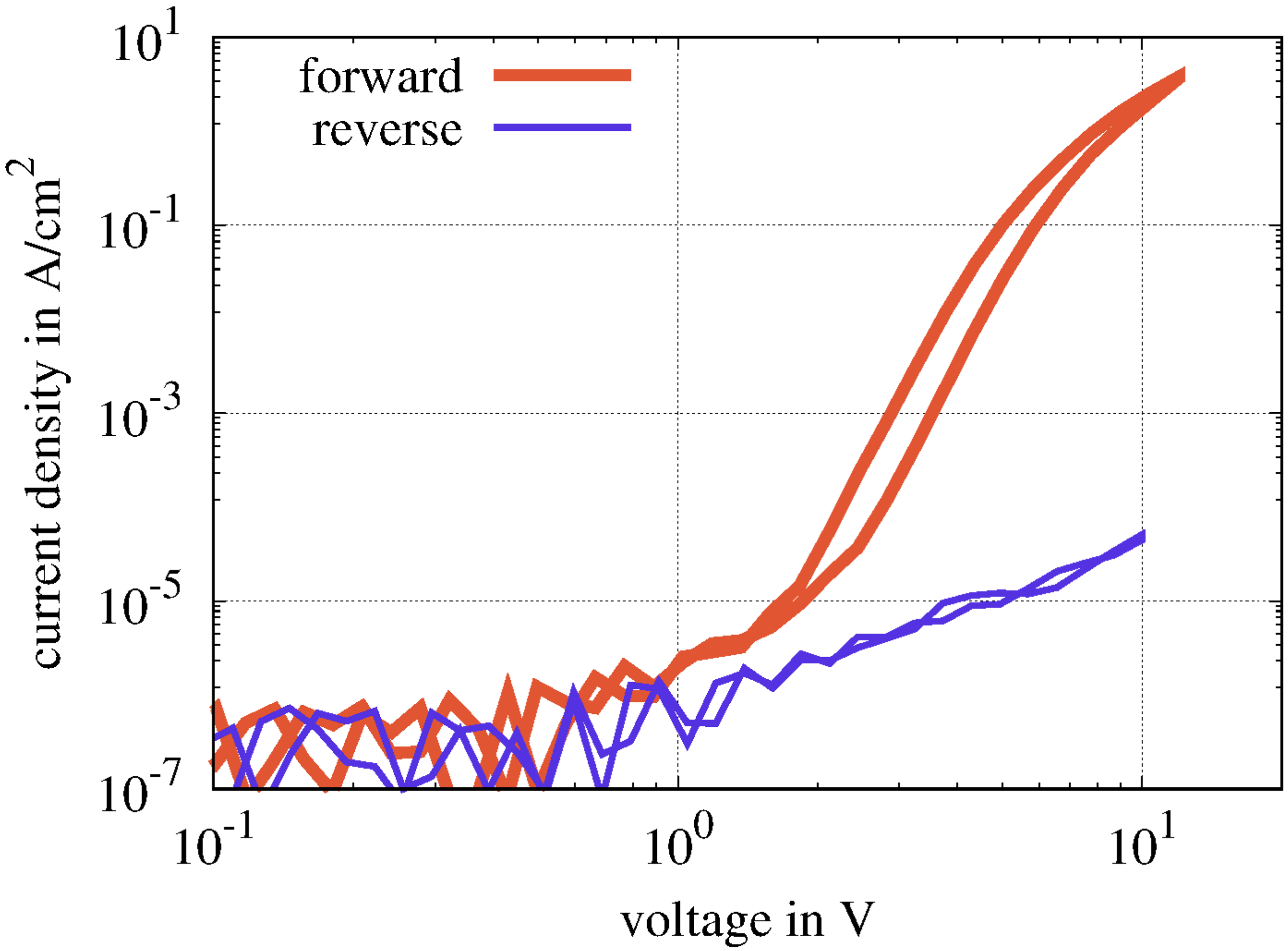

Figure 6: pin-diodes aimed at maximum on/off-ratio: Au-bottom electrode, 30 nm undoped seed, 300 nm rubrene doped with 5 wt.% F6-TCNNQ, 200 nm of intrinsic rubrene, 50 nm, of 0.5 wt.% $W_2(hpp)_4$, Al top-electrode.

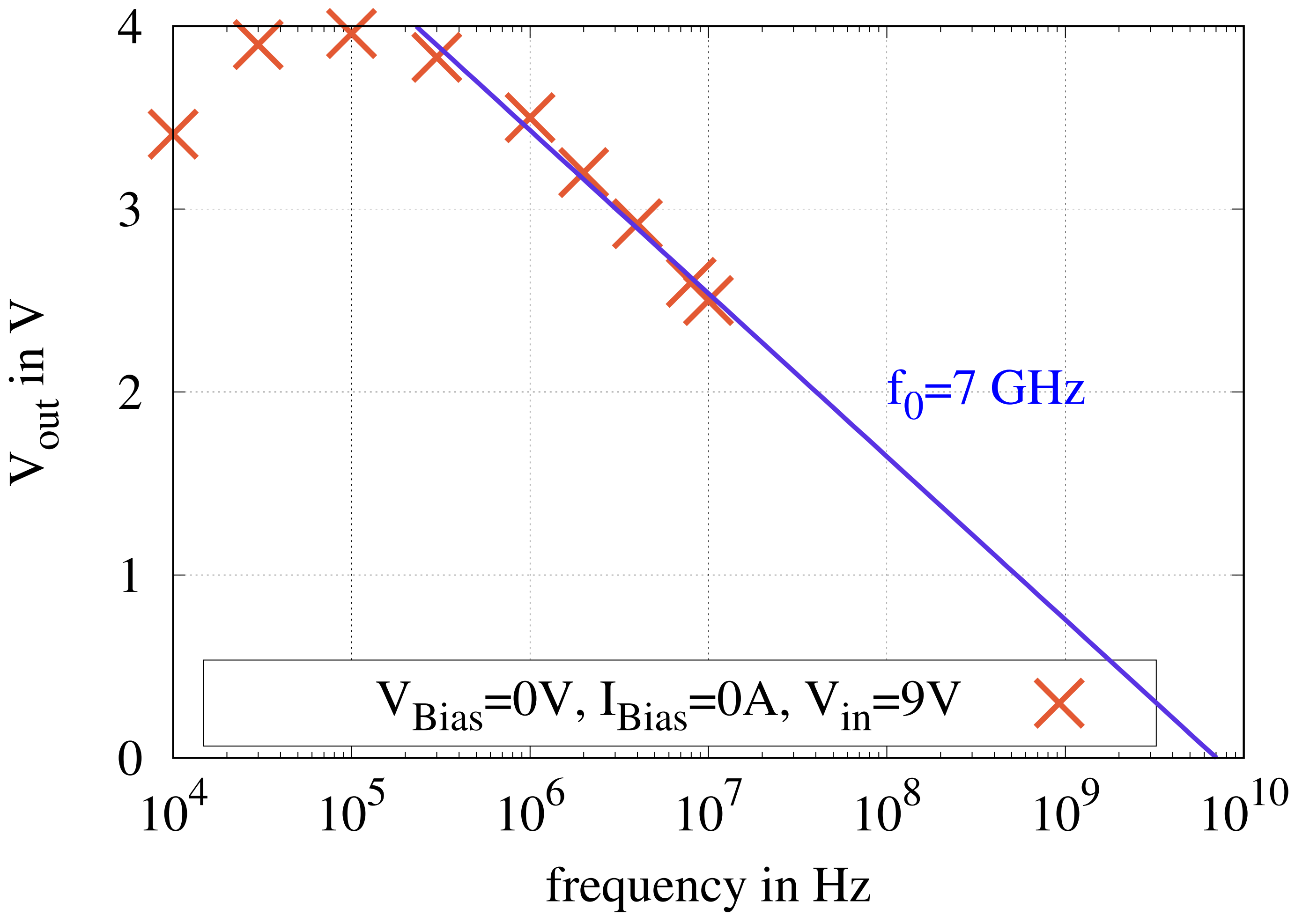

Figure 7: Rectified DC-output voltage as a function of frequency for a half-wave rectifier using a Schottky diode based on crystalline rubrene: R_L=1 MΩ, C_L =100 pF. Extrapolation of the decrease is added, suggesting a maximum rectification frequency f_0 of 7 GHz. Corresponding f_{3dB} =5.7 MHz.

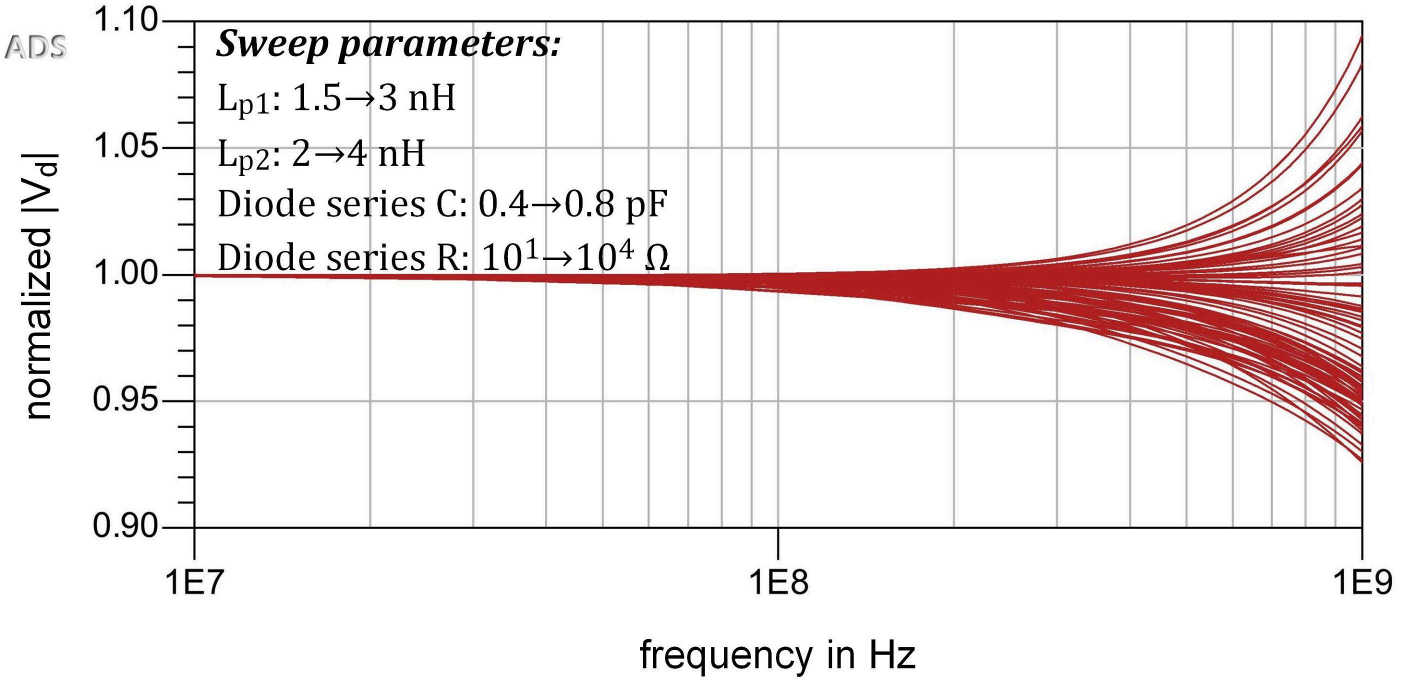

Figure 8: Possible variation of the input voltage at the diode V_d depending on the variation of circuit parameters; Simulated using Keysight ADS.

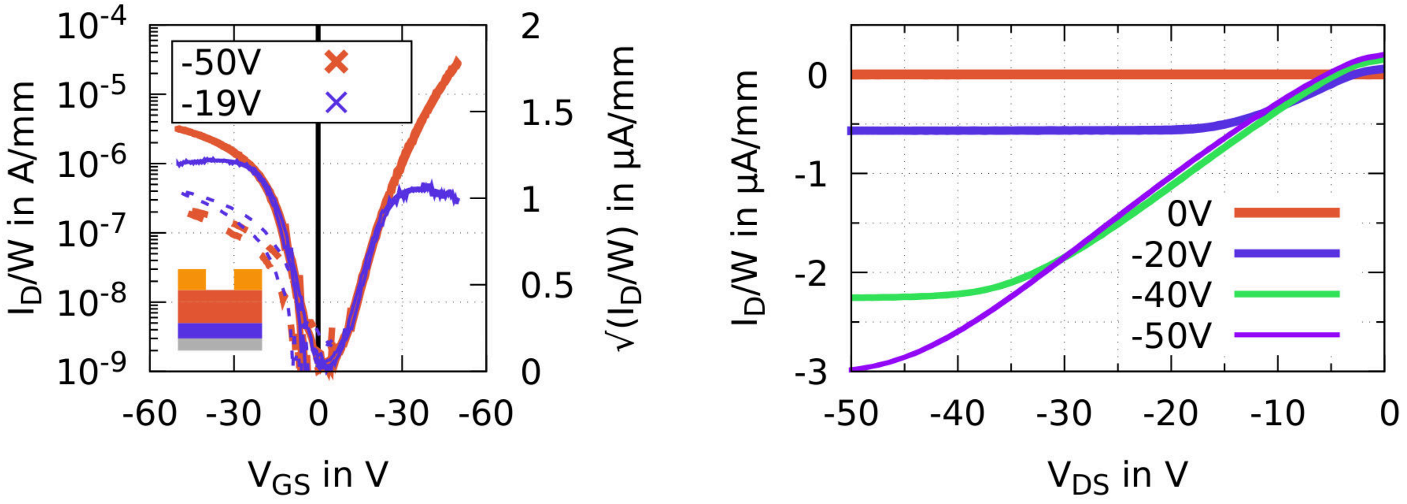

Figure 9: Transfer (left) and output (right) characteristics of a field-effect transistor based on triclinic rubrene in a bottom-gate, top-contact configuration. The substrate is p-doped Si (serving as the gate) with 100 nm of SiO_2 and 50 nm of Cytop deposited via spin-coating. Seed layer: 40 nm + 40 nm added rubrene via epitaxy to fill voids. Channel width: 1000 µm, channel length: 50 µm. (key: colors(drain-source voltage, solid lines: drain current, dashed lines: gate current)

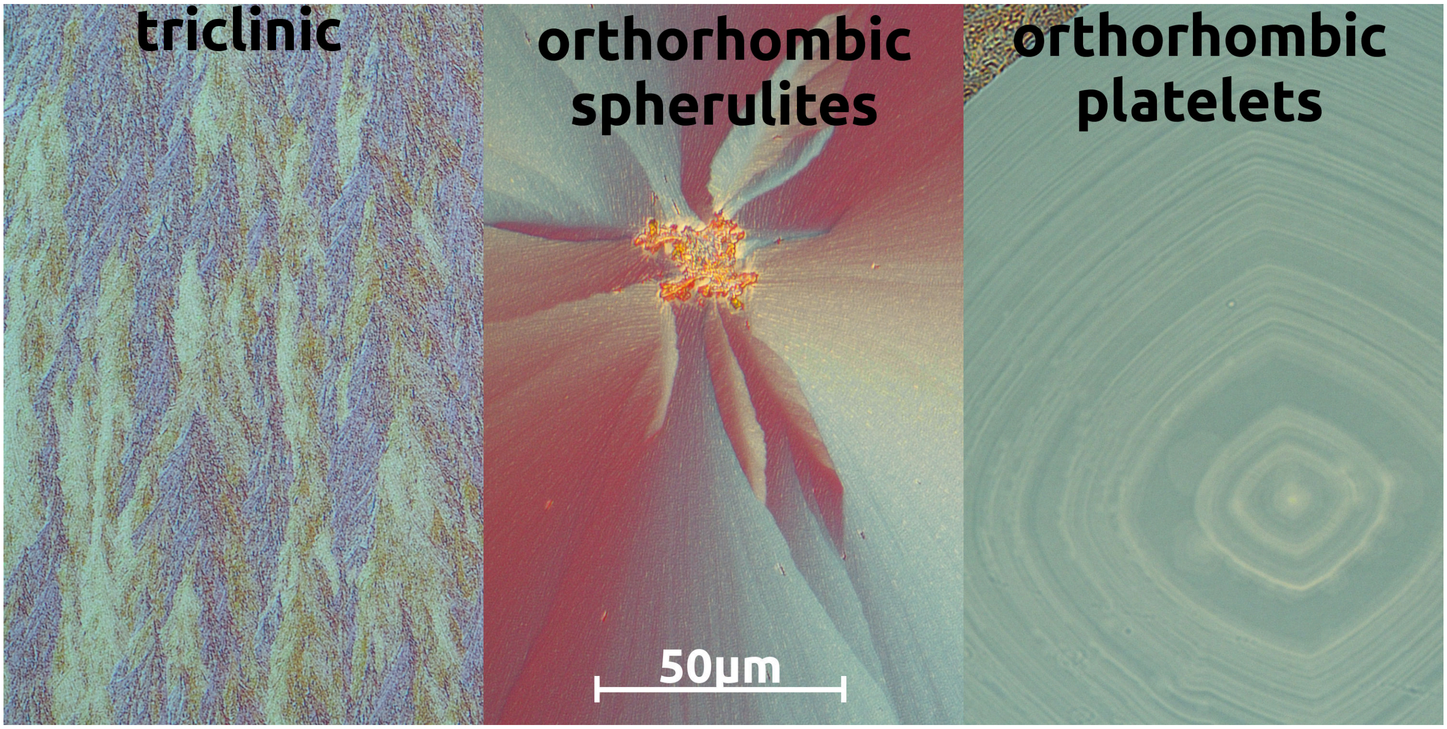

Figure 10: Polarized microscopy images of three different crystal thin-films phases of rubrene: triclinic (dendritic)-spherulites, orthorhombic Spherulites, and orthorhombic platelets.

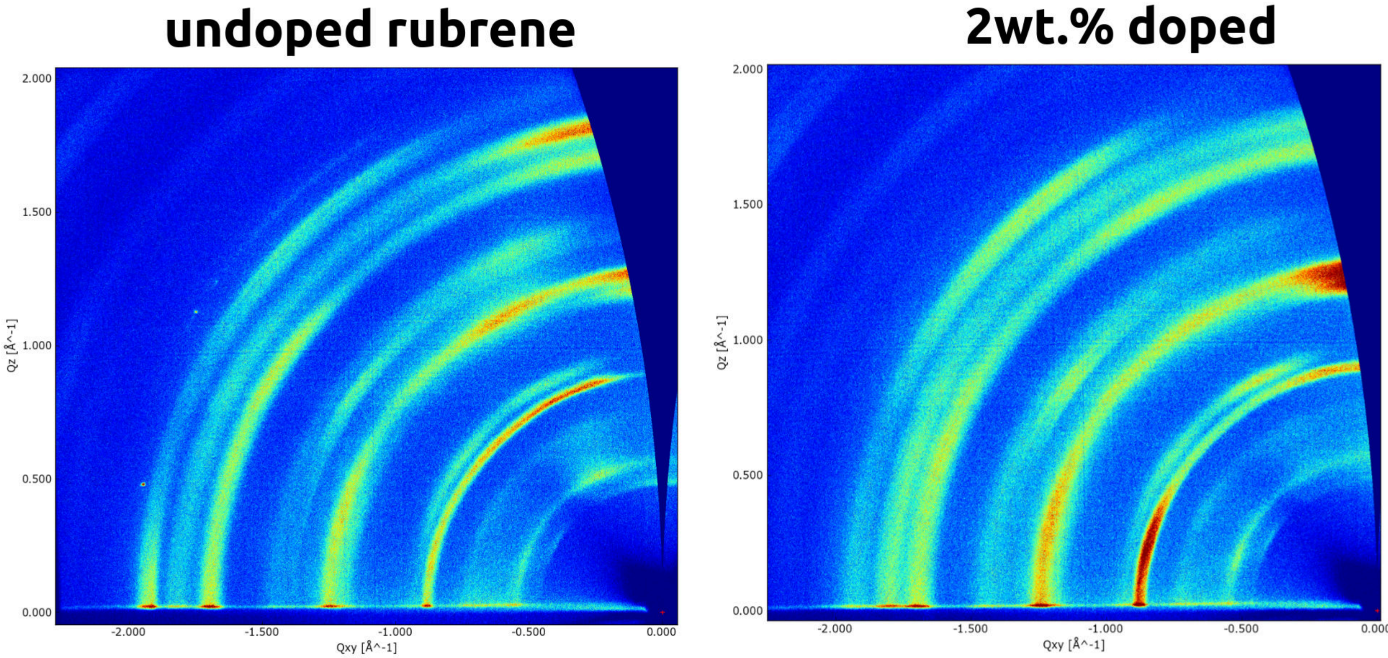

Figure 11: GIWAXS measurement of undoped and doped (2 wt.%) rubrene in the triclinic crystal phase on Si/SiO_2-substrates with a thickness of 100 nm

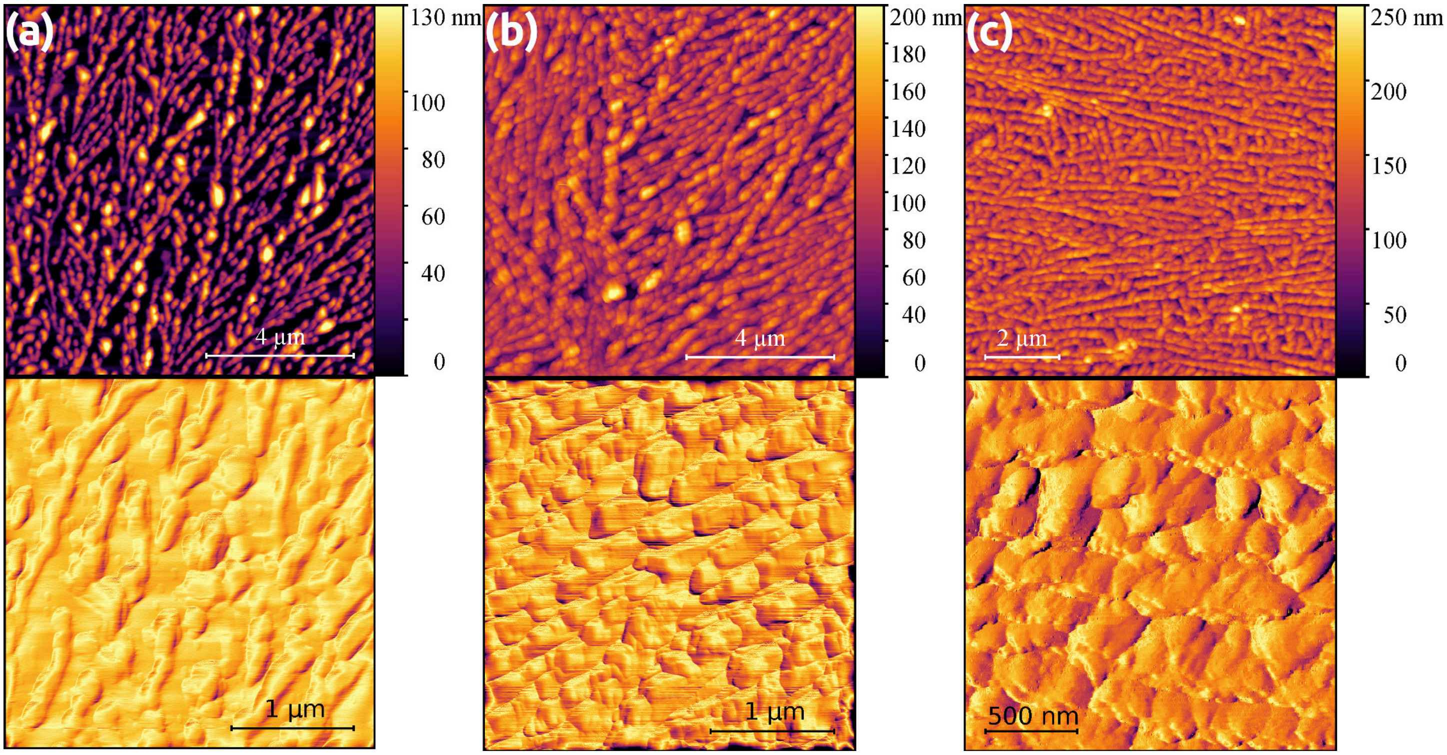

Figure 12: Atomic Force microscopy images of triclinic rubrene thin-films: **top:** height profile of seed only (a), seed + 40 nm intrinsic material (b), and entire diode stack (c), **bottom:** shaded depiction of fine-structure.

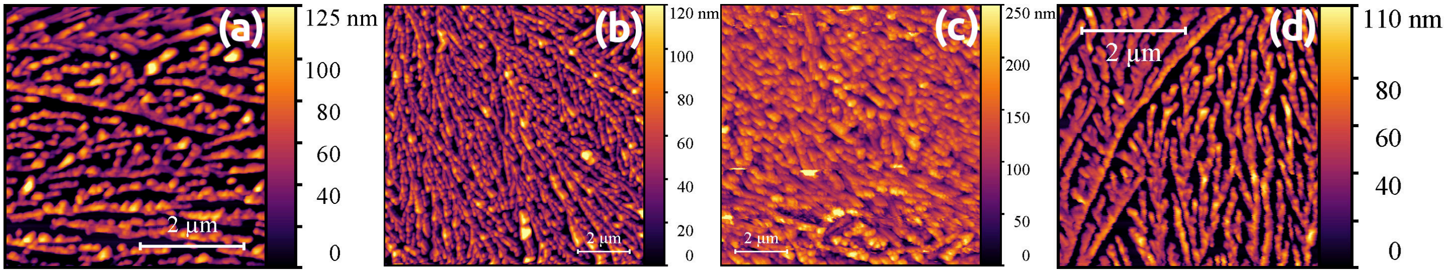

Figure 13: Atomic Force microscopy images of doped (F6-TCNNQ) triclinic rubrene thin-films: height profile of 0.5 wt.%-doped seed (a), 1 wt.%-doped seed (b), 1 wt.%-doped seed and 1 wt.%-doped 40 nm epitaxial layer (c), and 2 wt.%-doped seed.

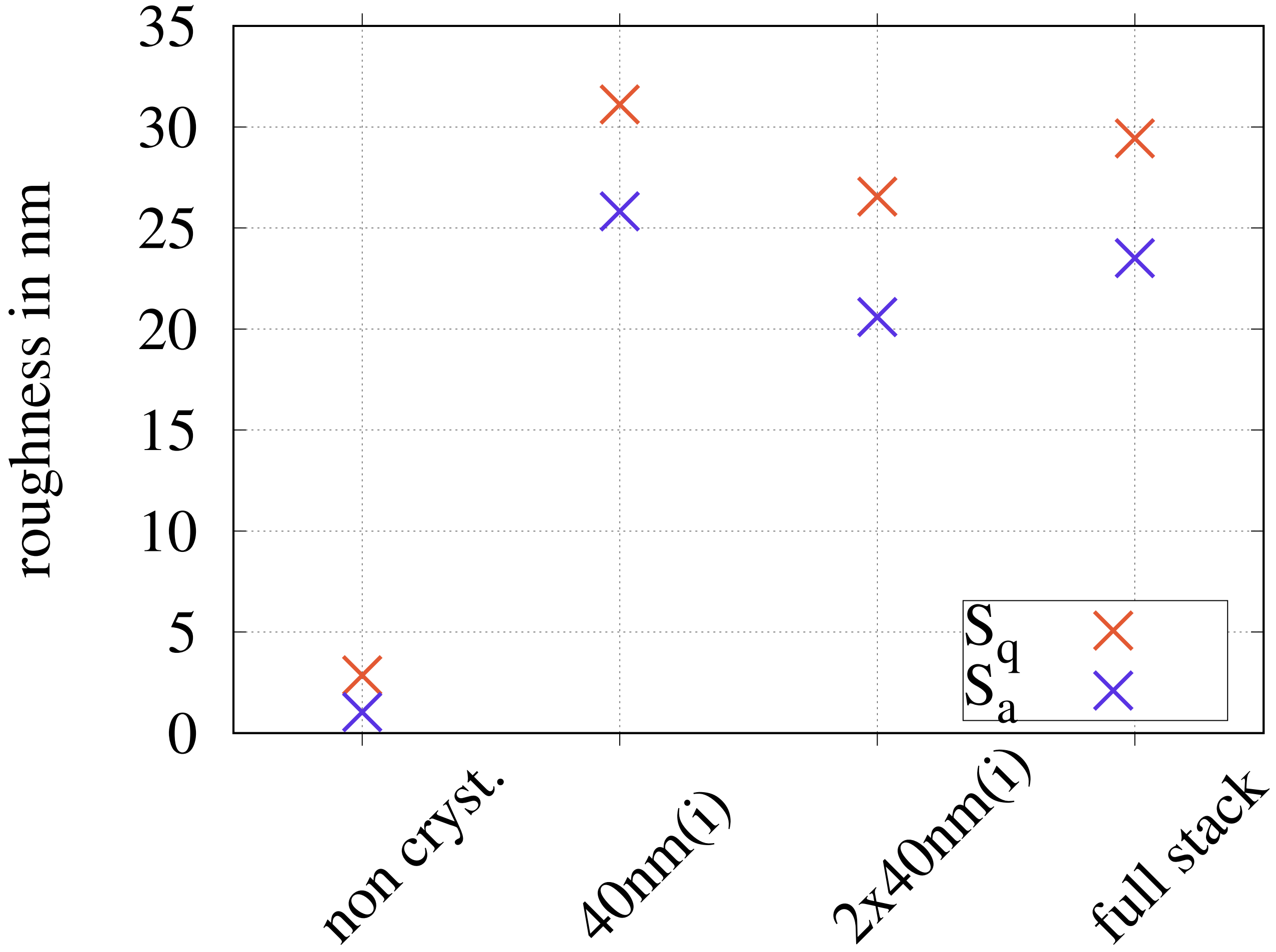

Figure 14: Average surface roughness (S_a) and average squared surface roughness (S_q) of AFM-measurements. The first data point is measured on uncrystallized amorphous rubrene. Further data points are based on SI Figure S12.

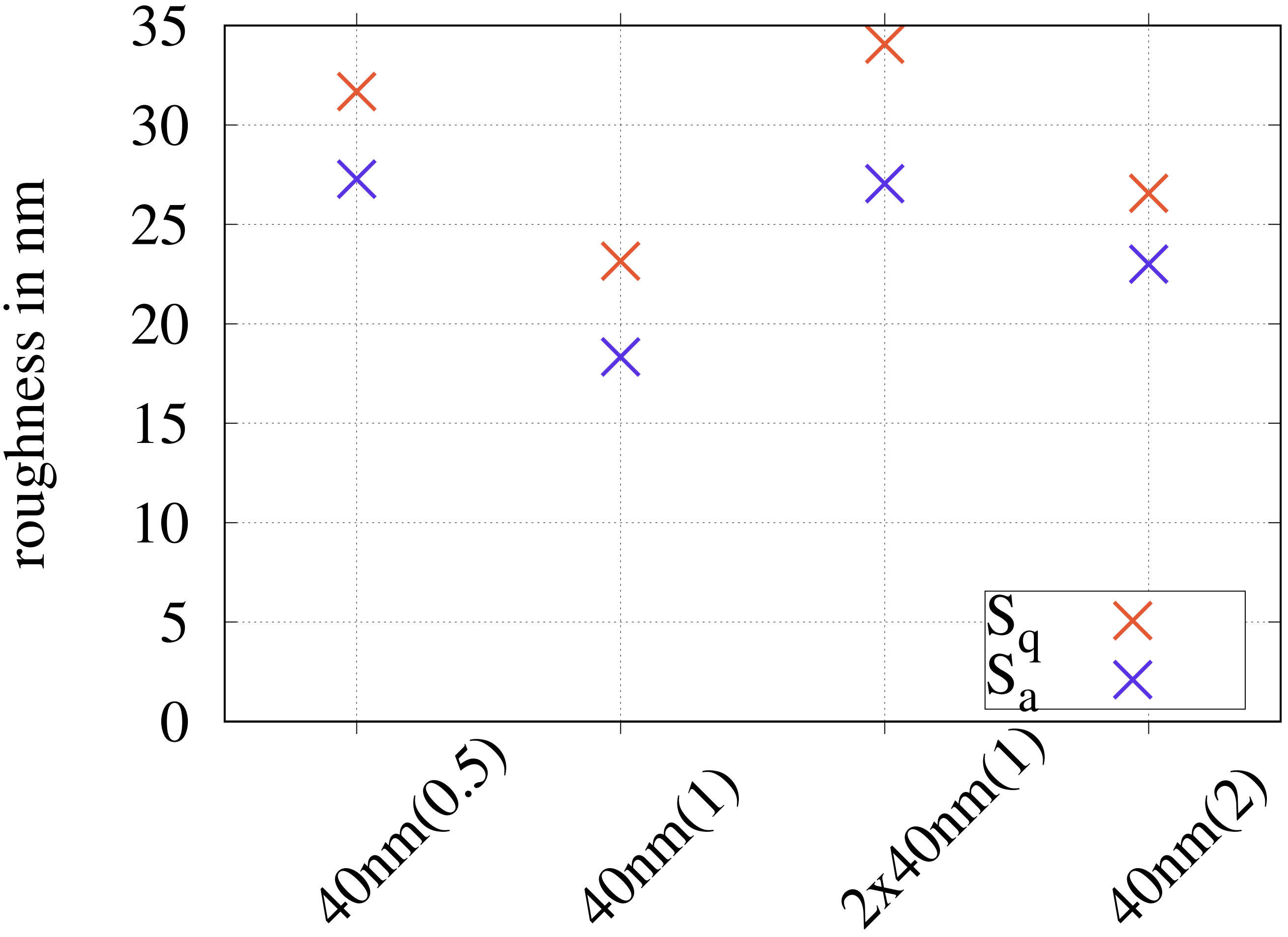

Figure 15: Average surface roughness (S_a) and average squared surface roughness (S_q) of AFM-measurements in SI Figure S13, doping concentration in wt.% in brackets.

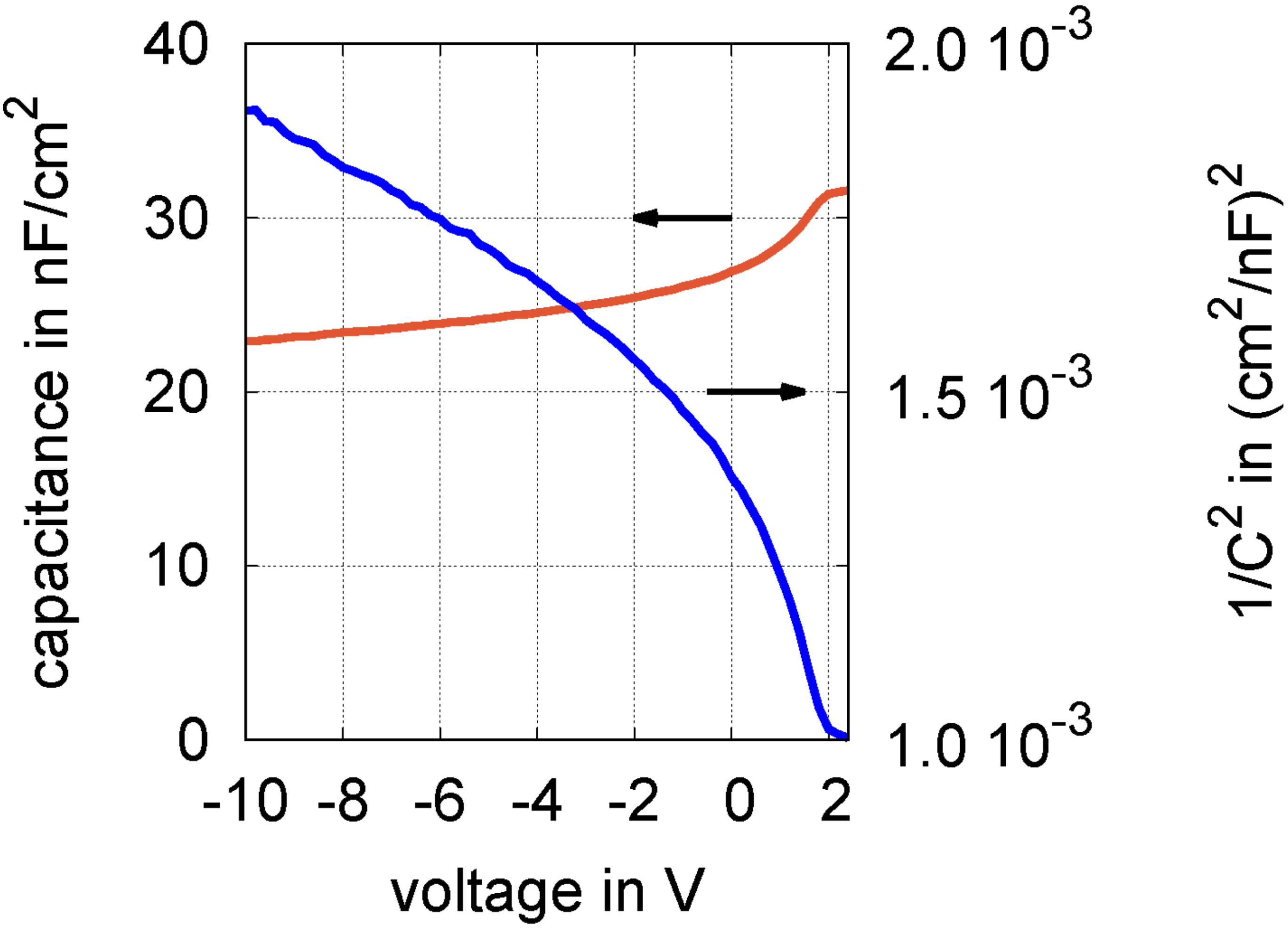

Figure 16: Capacitance-voltage-measurement (CV) at 10 kHz and 20 mV amplitude. Negative voltage indicates reverse bias. Active device area: 100 µm × 100 µm (The measurement is performed on a device outside of the rectifier circuit).

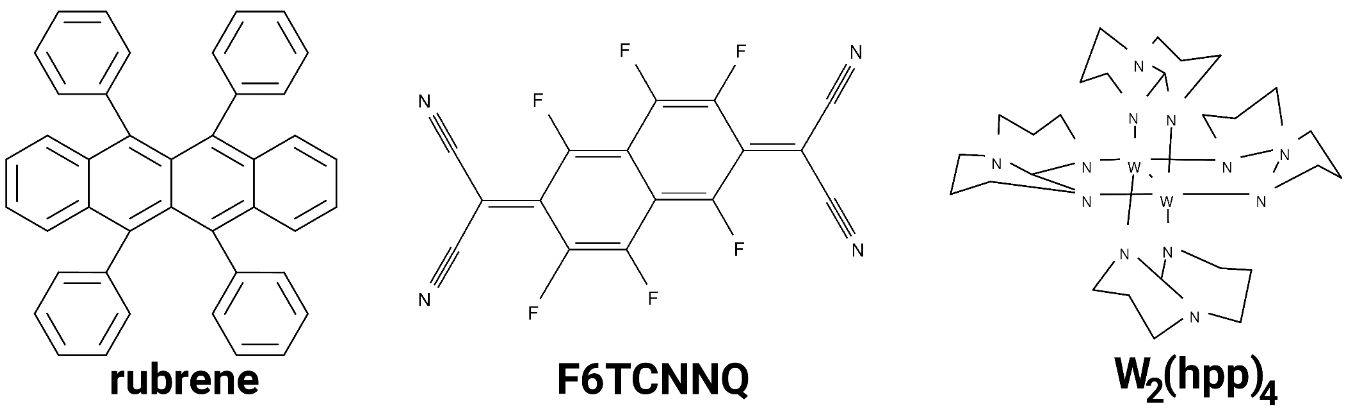

Figure 17: Rubrene is purchased from *TCI chemicals* in sublimed form and subsequently sublimed in-house a second time. F_6-TCNNQ (see [12] for details) and $W_2(hpp)_4$ (see [13] for details) are purchased from *Novaled GmbH*.
